# The frequency of mutations in the *pen*A, *mtr*R, *gyr*A and *par*C genes of *Neisseria gonorrhoeae*, the presence of *tet*M gene and antibiotic resistance/susceptibility: a systematic review and meta-analyses

**DOI:** 10.3389/fmicb.2024.1414330

**Published:** 2025-01-22

**Authors:** Ana Clara Mendes, Renan Pedra de Souza, Diana Bahia

**Affiliations:** Departamento de Genética, Ecologia e Evolução, Instituto de Ciências Biológicas, Universidade Federal de Minas Gerais, Belo Horizonte, Minas Gerais, Brazil

**Keywords:** *Neisseria gonorrhoeae*, antibiotic resistance, mutation, *pen*A, *mtr*R, *tet*M, *gyr*A, *par*C gene

## Abstract

Gonorrhoea is currently one of the most important sexually transmitted infections (STIs) due to the increasing spread of multidrug-resistant strains of *N. gonorrhoeae*. The aim of this study was to analyse the association between resistance or decreased susceptibility to antibiotics in *N. gonorrhoeae* and the presence of mutations in the *pen*A, *mtr*R, *gyr*A and *par*C genes, and the presence of tetM gene. We conducted a systematic review according to the PRISMA guidelines. We selected 19 studies for the *pen*A gene, 23 for *gyr*A and *par*C, 18 for *mtr*R and 12 for *tet*M using the Science Direct and PubMed databases. Meta-analyses of isolates resistant to penicillin, cefixime and ceftriaxone showed that more than 50% of isolates had mutations in the *pen*A and *mtr*R genes. More than 50% of azithromycin-resistant isolates had mutations in the *mtr*R gene, while more than 50% of ciprofloxacin-resistant and intermediate-resistant isolates had mutations in *gyr*A. Less than 50% of the isolates with intermediate resistance to ciprofloxacin had mutations in *par*C. The plasmid containing the *tet*M gene was found in more than 50% of tetracycline-resistant isolates. Infection surveillance and genetic studies are important for controlling the spread of the disease, which can improve the quality of life of infected people and reduce the financial burden on public health systems.

## 1 Introduction

Sexually transmitted infections (STIs) are one of the main global public health problems and among the main transmissible diseases, affecting the health of men, women, and infants around the world (World Health Organization, 2019b). It is estimated that, worldwide, over 1 million new STI cases occur daily (376.4 million new infections annually) due to the four currently treatable STIs: chlamydia, gonorrhoea, syphilis, and trichomoniasis (World Health Organization, 2019a). On average, approximately 1 in 25 people has at least one STI. Since the last published data, there has been no significant decline in existing or new infections (World Health Organization, 2019b).

Gonorrhoea is an STI that exclusively affects humans and is caused by the bacterium *Neisseria gonorrhoeae*, a Gram-negative diplococcus, also known as gonococcus (Quillin and Seifert, 2018). The infection causes urethritis in males and cervicitis in females. STIs, if unidentified or untreated, can ascend in the genital tract and result in complications (Rice et al., 2017). In addition, the infection increases the rates of transmission and acquisition of HIV (Cohen et al., 1996). In 2020, the WHO estimated that there were 82.4 million cases of gonorrhoea worldwide (World Health Organization, 2024). Sexual orientation, sexual behavior, socioeconomic status, geographical location, culture, and access to sex education are directly related to the epidemiological variety of the disease (World Health Organization, 2019a,2019b).

Gonorrhoea is currently one of the most important STIs due to the increase in the spread and emergence of multidrug-resistant strains; it is also the second most common STI globally (Unemo et al., 2019). For this reason, the WHO has included the bacterium *N. gonorrhoeae* in the list of priorities for combatting transmission and researching new drugs (World Health Organization, 2017). Currently, *N. gonorrhoeae* is resistant to penicillin, tetracycline, macrolides, azithromycin, sulphonamides, quinolones and cephalosporins such as cefixime and ceftriaxone. Many cephalosporin-resistant strains are also resistant to other antibiotics, making *N. gonorrhoeae* a multidrug-resistant bacterium (Unemo and Shafer, 2014; World Health Organization, 2017).

*Neisseria gonorrhoeae* can change its genetic material through several types of mutations, which it uses to adapt and survive in the human host. This species has evolved and acquired or developed mechanisms of resistance to almost all types of antibiotics recommended for use in treatment (Unemo and Shafer, 2014). In addition, *N. gonorrhoeae* is naturally competent for transformation, referring to the ability to take up and incorporate DNA, which occurs via DNA donation, DNA binding and uptake, processing and homologous recombination. Transformation occurs particularly often between *N. gonorrhoeae* and other *Neisseria* species and is an important mechanism to generate genetic diversity. It has also been shown that commensal *Neisseria* is a reservoir of genetic material for pathogenic *Neisseria* (Rotman and Seifert, 2014; Quillin and Seifert, 2018; Unemo et al., 2019). The main genes related to antibiotic resistance of *N. gonorrhoeae* are *pen*A, *bla*TEM, *mtr*R, *tet*M, *gyr*A and *par*C (Unemo et al., 2016).

Beta-lactam antibiotics, such as penicillin and cephalosporins, inhibit peptidoglycan synthesis in the bacterial cell wall by binding the beta-lactam ring to transpeptidase enzymes called penicillin-binding proteins (PBPs) located in the periplasm. Resistance results from cumulative chromosomal mutations in genes involved in cell wall synthesis, such as *pen*A. Mutations in the *pen*A gene alter the PBP protein, preventing antibiotic binding. *pen*A allelic mosaics are associated with reduced susceptibility to cephalosporins. These alleles in *pen*A are called “mosaics” because their DNA sequence appears to have been formed by homologous recombination with DNA from other species of *Neisseria* that are naturally resistant to third-generation cephalosporins (Gose et al., 2013).

Tetracyclines inhibit the binding of aminoacyl-tRNA to the mRNA-ribosome complex by binding to the 30S ribosomal subunit, thereby inhibiting protein synthesis. The *tet*M gene, carried by plasmids in *N. gonorrhoeae*, confers resistance to tetracyclines because it encodes the *tet*M protein, which binds to the ribosome and protects it from antibiotic binding, allowing the process of protein synthesis (Morse et al., 1986; Wi et al., 2017).

Quinolones/fluoroquinolones, such as ciprofloxacin, inhibit the activity of DNA gyrase and topoisomerase IV, enzymes essential for DNA replication, transcription, recombination and repair in bacteria. This class of antibiotics acts by forming a drug-enzyme-DNA complex, causing double-strand breaks in the DNA (Costa-Lourenço et al., 2017). Gonococcal resistance to ciprofloxacin is mediated by mutations in the quinolone resistance-determining region (QRDR). Mutations in the DNA gyrase enzyme (encoded by the *gyr*A gene) and topoisomerase IV (encoded by the *par*C gene) reduce the binding of the antibiotic to these enzymes and prevent its action (Belland et al., 1994; Sánchez-Busó et al., 2019; Wi et al., 2017).

Mtr (multiple transferable resistance) efflux pumps are triple efflux pumps (MtrCDE) that export a variety of antimicrobial agents such as antimicrobial peptides, antibiotics, bile salts and fatty acids. MtrCDE, whose expression is controlled by the *mtr*R repressor, is composed of inner membrane (MtrD) and outer membrane (MtrE) channels connected by a periplasmic membrane fusion lipoprotein (MtrC) (Handing et al., 2018). These efflux pumps control the concentration of drugs in the periplasm and regulate the efflux of antibiotics such as tetracyclines, penicillins, quinolones, cephalosporins and macrolides (Cousin et al., 2003; Unemo and Shafer, 2014). Mutations in *mtr*R, in the promoter or coding sequence, promote overexpression and increased efflux of the MtrCDE efflux pump, leading to resistance to these antibiotics (Costa-Lourenço et al., 2017).

In reviewing the antimicrobial resistance mechanisms of *N. gonorrhoeae*, Unemo and Shafer (2014) suggested that the world may be entering an era of intractable *N. gonorrhoeae* due to multi-drug resistance. Antibiotic-resistant *N. gonorrhoeae* can appear as a silent epidemic, and the disease and its complications can cause morbidity and economic consequences, as treatment can become more expensive when complications occur.

Several studies, such as those by Lee et al., (2015) and Calado et al., (2019), evaluated the minimum inhibitory concentrations (MICs) of *N. gonorrhoeae* isolates and searched for *N. gonorrhoeae* resistance genes and resistance gene mutations of resistant and/or reduced susceptibility isolates. They also correlated the mutations or presence of resistance genes with resistance or reduced susceptibility to antibiotics. Building on these previous studies, this work involved a systematic review aimed at identifying critical articles focusing on these characteristics and selecting appropriate ones for analysis using a set of criteria. The meta-analysis performed here should deepen our understanding of gonorrhoea and help develop solutions to this urgent public health problem.

## 2 Methods

This systematic review was conducted considering the protocol proposed by PRISMA (Moher et al., 2009). The phases for the selection of studies were identification, selection, eligibility and inclusion (Table 1, see also Figure 7). The search focused on the databases PubMed and Science Direct, including all articles within them published to date. The first screening was conducted based on reading of the title and abstract, after which the eligibility was determined through complete reading of the article, with this work being conducted by two researchers independently. This review includes studies on the *N. gonorrhoeae* genes *pen*A, *mtr*R, *tet*M, *par*C and *gyr*A, which are related to resistance, intermediate resistance or reduced susceptibility to penicillin, cefixime, ceftriaxone, azithromycin, ciprofloxacin and tetracycline.

**Table 1:** Phases of the studies selection, see also Figure 7.

	Identification		Screening			
Gene	Identification from databases	Screened	Excluded	Assessed for eligibility	Excluded	Studies included in qualitative and quantitative analysis
*pen*A	226	62	164	19	43	19
*gyr*A and *par*C	185	53	132	23	30	23
*mtr*R	180	66	114	18	48	18
*tet*M	102	26	76	12	14	12

The gene *pen*A (*Pen*A *family class A beta-lactamase*) is related to resistance to penicillins and cephalosporins. The genes *gyr*A (*DNA gyrase subunit A*) and *par*C (*DNA topoisomerase IV subunit A*) are related to resistance to quinolones, the *mtr*R (*multidrug efflux system transcriptional repressor*) gene is related to penicillin, tetracycline, cephalosporins and macrolides, and the gene *tet*M (*tetracycline resistance ribosomal protection protein*) is related to resistance to tetracyclines.

### 2.1 Search argument

For the PubMed search, the following search argument was used: [“Gonorrhea”(Mesh) OR “Gonorrhea”(Title/Abstract) OR “*Neisseria gonorrhoeae*”(Mesh) OR “*Neisseria gonorrhoeae*”(Title/Abstract) OR “*N. gonorrhoeae*”(Title/Abstract)] AND [“Antimicrobial resistance”(Title/Abstract) OR “Antimicrobial susceptibility”(Title/Abstract) OR “Resistance Profile”(Title/Abstract) OR “Drug Resistance, Microbial”(Mesh)] AND [“penA”(Title/Abstract)]. The argument was adapted for each gene.

For the search in the Science Direct database, the keywords “*Neisseria gonorrhoeae*, Antimicrobial resistance, *pen*A, *mtr*R, *tet*M, *gyr*A and *par*C” were used. In terms of the manuscript type, the search was limited to research articles.

For the articles, no restrictions on the year or site of publication were applied, while the inclusion criteria were articles in either Portuguese or English that address susceptibility and resistance to antibiotics and mutations in the selected genes. The exclusion criteria were articles in languages other than English and Portuguese, articles about other *Neisseria* species, studies that did not analyze antibiotic susceptibility profiles, case reports, review articles, studies that analyzed the application of a technique, articles that detected susceptibility and mutations in non-clinical isolates and articles with incomplete genotypic data.

### 2.2 Meta-analysis

Meta-analysis was conducted when two or more studies were found for the same gene and the same antibiotic. For this, the metaprop function of the meta package was used in the R program (version 4.0.2). The original proportions were combined using the inverse variance method with both fixed- and random-effects models. The fixed-effects model assumes that all studies estimate the same underlying effect size, meaning that any observed differences between study results are due solely to chance. In contrast, the random-effects model assumes that the true effect size may vary between studies due to differences in study populations, methodologies or other factors, reflecting real variations in effect size. This model incorporates both within-study variance and an additional between-study variance component to account for this heterogeneity (Dettori et al., 2022). Heterogeneity across studies was assessed using the *I*^2^ statistic, which represents the percentage of total variation attributable to heterogeneity, and tau^2^, which quantifies the absolute between-study variance. The significance of heterogeneity was evaluated using Cochran’s Q test. A significance level of 5% was set for all analyses.

## 3 Results

### 3.1 Selection of studies

The screening and selection processes are summarized in the are summarized in Table 1 and Figure 7.

### 3.2 Studies’ characteristics

The studies’ characteristics are summarized in Supplementary Tables S1–S6 according to the phenotypes. In general, we observed many common characteristics across studies. The earliest included studies investigating antibiotic resistance, decreased susceptibility and mutations in resistance genes were performed in 1998. Research was conducted in various countries, but particularly in East Asia, mainly China and Japan (Supplementary Tables S1–S6, column “Local”). In most of the studies, samples were collected from urethra, rectum, cervix, endocervix and pharynx; in only one study were samples collected from other sites such as eye, blood, surgical wound, gastric juice, synovial fluid and Bartholin abscess. Moreover, the results show a lack of studies in Latin America and Africa. Many papers do not provide details on clinical isolates beyond the study period (second column). Although fewer in number, some papers detail sex, sexuality and sexual behavior. In studies providing information on sex, most of the isolates were obtained from samples collected from men.

The papers also reveal which mutations were found most frequently (Supplementary Tables S7–S10). The most common mutations in the *pen*A gene were A501V substitution and mosaic-type mutations. In the *mtr*R gene, deletion of A (adenine), G45D, H105Y and A39T substitutions were the most common mutations. Meanwhile, the *gyr*A gene presented the S91F and D95G substitutions and the *par*C gene the D86N substitution.

### 3.3 Meta-analysis of studies evaluating penicillin resistance

The results of the meta-analysis for the antibiotic penicillin with the proportions of mutations in the *pen*A and *mtr*R genes are shown in Supplementary Figure S1. The results of the random-effects model indicate that 87% (CI 42–98%) of the analyzed isolates had mutations in the *pen*A gene (Supplementary Figure 1A). Fixed-effect model results indicate that 95% (CI 86–98%) had mutations in the *mtr*R gene (Supplementary Figure 1B).

The meta-analysis results of studies on the antibiotic penicillin with the proportions of mutations in the *mtr*R gene are shown in Supplementary Figure S2. The results of the fixed model indicate that 85% (CI 71–93%) of the analyzed isolates had mutations in the *mtr*R gene.

### 3.4 Meta-analysis of studies evaluating resistance and reduced susceptibility to cefixime

Supplementary Figure S3 presents the meta-analysis results of studies on cefixime resistance and the proportions of mutations in the *mtr*R and *pen*A genes. The results of the fixed-effects model indicate that 94% (CI 65–99%) of the analyzed isolates had mutations in the *mtr*R gene (Supplementary Figure 3A) and 93% (CI 77–98%) of them had mutations in the *pen*A gene (Supplementary Figure 3B).

Supplementary Figure S4 presents the meta-analysis results of studies on reduced susceptibility to cefixime. The results of the random-effects model indicate that 93% (CI 73–99%) of the analyzed isolates had mutations in the gene *pen*A (Supplementary Figure 4A). Meanwhile, the results of the fixed-effects model indicate that 96% (CI 88–99%) of the isolates had mutations in the *mtr*R gene (Supplementary Figure 4B).

Supplementary Figures S5, S6 present the results of the meta-analysis of the systematic review of studies on the *mtr*R gene. Supplementary Figure S5 presents the results of the meta-analysis of studies on reduced susceptibility to the antibiotic cefixime with the proportions of mutations in the *mtr*R gene. The results of the fixed-effects model show that 96% (CI 88–99%) of the analyzed isolates had mutations in the *mtr*R gene. Supplementary Figure S6 presents the result of the meta-analysis of cefixime-resistant isolates with the proportions of mutations in the *mtr*R gene. The results of the fixed-effects model indicate that 94% (CI 65–99%) of the analyzed isolates had mutations in the *mtr*R gene.

### 3.5 Meta-analysis of studies evaluating resistance and reduced susceptibility to ceftriaxone

Supplementary Figure S7 presents the meta-analysis results on ceftriaxone resistance with the proportions of mutations in the *pen*A (Supplementary Figure 7A) and *mtr*R (Supplementary Figure 7B) genes. The results of the fixed-effects model show that 93% (CI 82–97%) of the analyzed isolates had mutations in the *pen*A gene (Supplementary Figure 7A), and 90% (CI 72–97%) of the isolates had mutations in the *mtr*R gene (Supplementary Figure 7B).

Supplementary Figure S8 shows the meta-analysis results of studies on reduced susceptibility to ceftriaxone with the proportions of mutations in the *pen*A (Supplementary Figure 8A) and *mtr*R (Supplementary Figure 8B) genes. The results of the random-effects models indicate that 95% (CI 85–99%) of the isolates had mutations in the *pen*A gene (Supplementary Figure 8A) and 94% (CI 80–99%) of the isolates had mutations in the *mtr*R gene (Supplementary Figure 8B).

Supplementary Figures S9, S10 present the meta-analysis results of the systematic review of the *mtr*R gene. Supplementary Figure S9 presents the meta-analysis results of studies on reduced susceptibility to ceftriaxone with the proportions of mutations in the *mtr*R gene. Results from the random-effects model indicate that 71% (CI 58–82%) of the isolates had mutations in the *mtr*R gene.

Supplementary Figure S10 presents the meta-analysis results of studies on ceftriaxone resistance with the proportions of *mtr*R mutations. Fixed-effects model results show that 93% (CI 72–99%) of the isolates had mutations in the *mtr*R gene.

### 3.6 Meta-analysis of studies evaluating resistance to azithromycin

**Figure 1:**
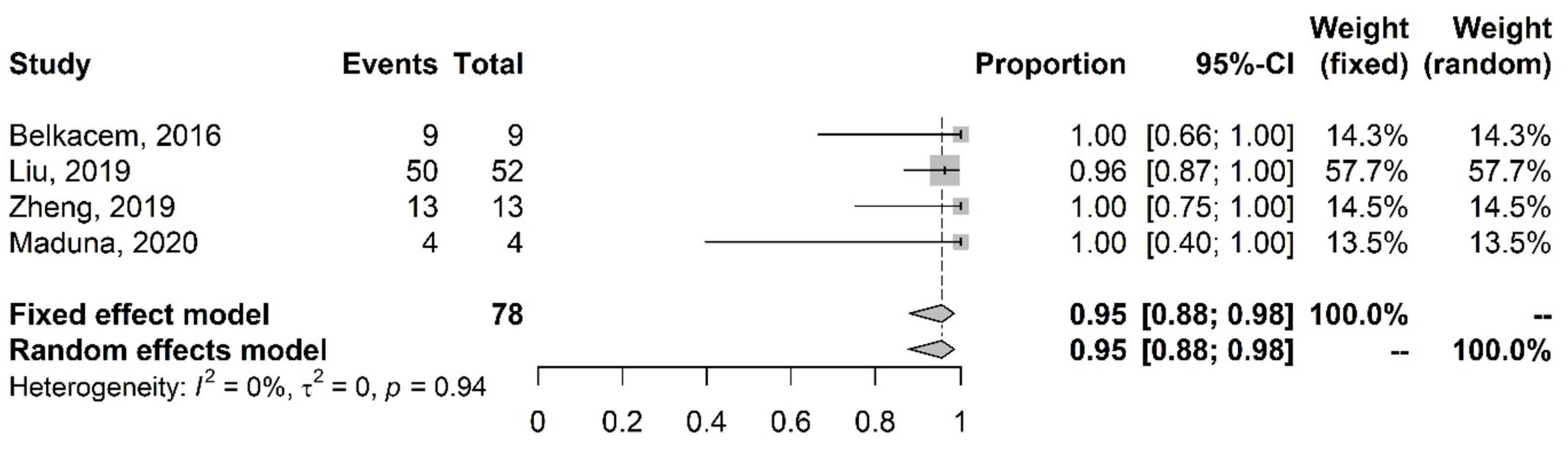
Meta-analysis of the proportion of gene mutations in azithromycin-resistant isolates (*mtr*R gene). The results are presented in forest plots. In its first column (“study”) are the citations of the selected studies. The second column (“events”) contains the number of isolates with mutations in the evaluated gene, while the third column (“total”) shows the total number of samples analyzed in that study. The fourth column presents the proportion estimate followed by their confidence intervals. The sixth and seventh columns present, respectively, the weights for the fixed and random models of the meta-analysis.

The meta-analysis results of studies of azithromycin-resistant isolates with the proportions of mutations in the *mtr*R gene are shown in Figure 1. The results of the fixed-effects model show that 95% (CI 88–98%) of the analyzed isolates had mutations in the *mtr*R gene.

### 3.7 Meta-analysis of studies evaluating resistance and intermediate resistance to ciprofloxacin

**Figure 2:**
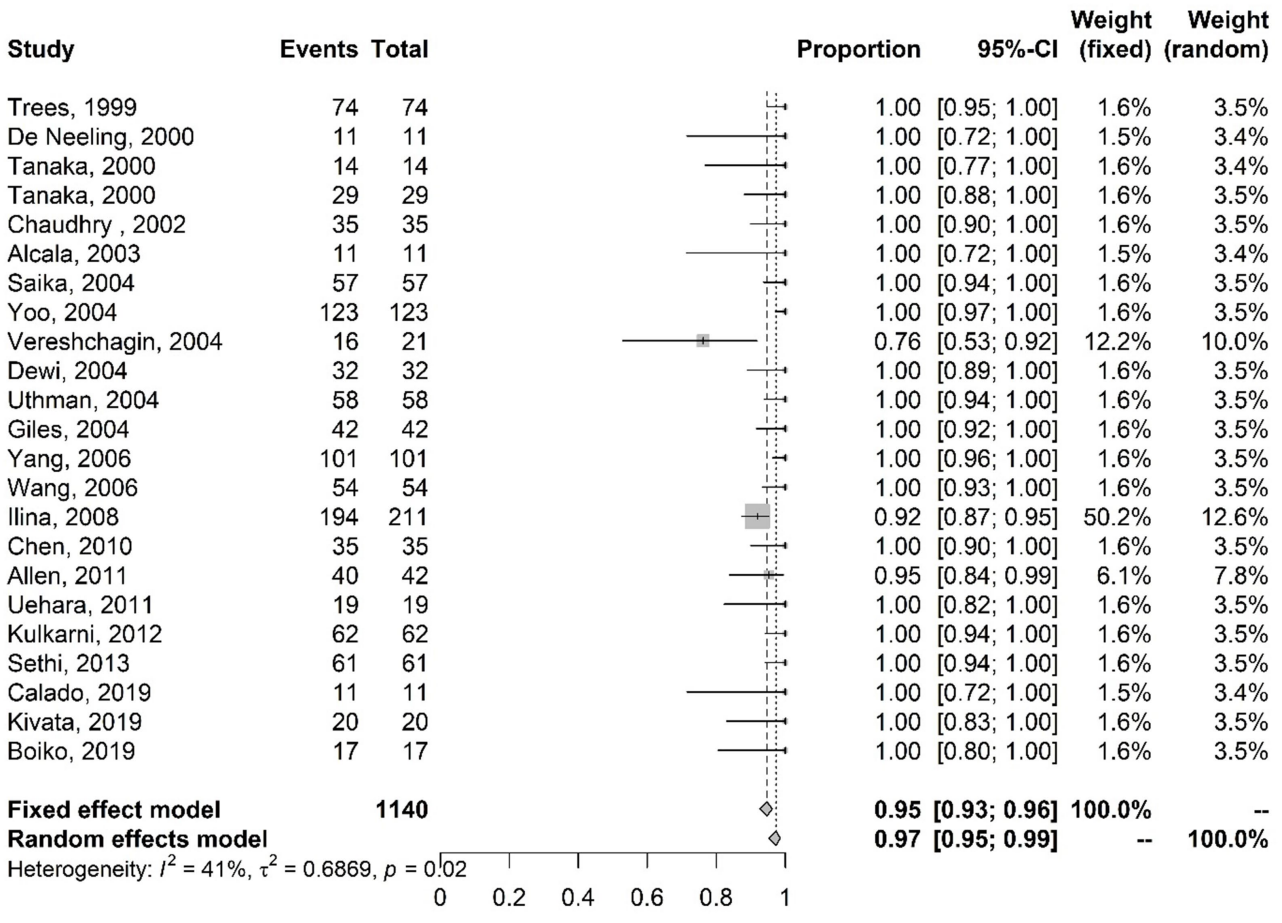
Meta-analysis of the proportion of gene mutations in isolates resistant to ciprofloxacin (*gyr*A gene). The results are presented in forest plots. In its first column (“study”) are the citations of the selected studies. The second column (“events”) contains the number of isolates with mutations in the evaluated gene, while the third column (“total”) shows the total number of samples analyzed in that study. The fourth column presents the proportion estimate followed by their confidence intervals. The sixth and seventh columns present, respectively, the weights for the fixed and random models of the meta-analysis.

The meta-analysis results of studies on ciprofloxacin resistance with the proportions of mutations in the *gyr*A gene are shown in Figure 2. The results of the random-effects model show that 97% (95–99% CI) of the isolates had mutations in the *gyr*A gene.

**Figure 3:**
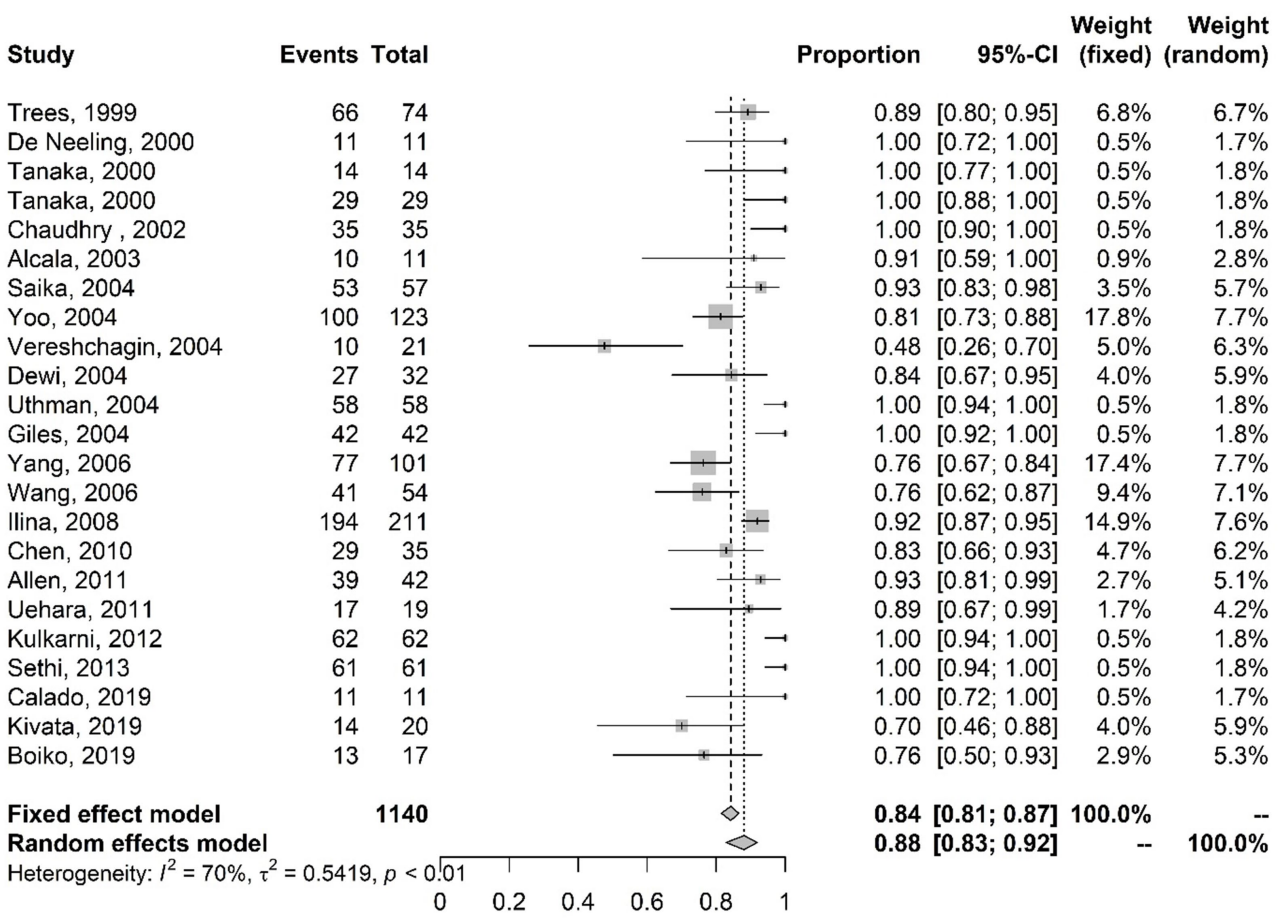
Meta-analysis of the proportion of gene mutations in isolates resistant to ciprofloxacin (*par*C gene). The results are presented in forest plots. In its first column (“study”) are the citations of the selected studies. The second column (“events”) contains the number of isolates with mutations in the evaluated gene, while the third column (“total”) shows the total number of samples analyzed in that study. The fourth column presents the proportion estimate followed by their confidence intervals. The sixth and seventh columns present, respectively, the weights for the fixed and random models of the meta-analysis.

Figure 3 presents the meta-analysis results of studies on ciprofloxacin resistance with the proportions of mutations in the *par*C gene. The results of the random-effects model indicate that 88% (CI 83–92%) of the analyzed isolates have mutations in the *par*C gene.

**Figure 4:**
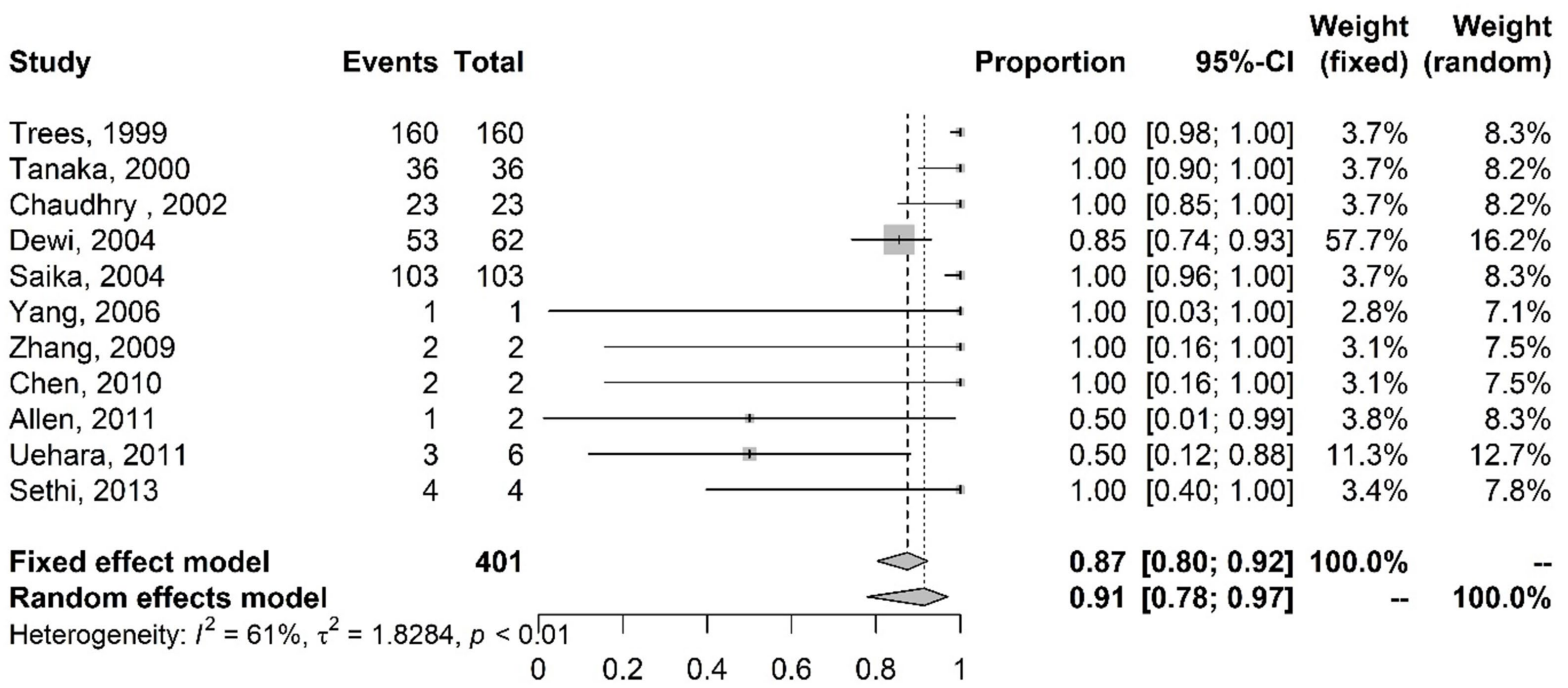
Meta-analysis of the proportion of gene mutations in isolates with intermediate resistance to ciprofloxacin (*gyr*A gene). The results are presented in forest plots. In its first column (“study”) are the citations of the selected studies. The second column (“events”) contains the number of isolates with mutations in the evaluated gene, while the third column (“total”) shows the total number of samples analyzed in that study. The fourth column presents the proportion estimate followed by their confidence intervals. The sixth and seventh columns present, respectively, the weights for the fixed and random models of the meta-analysis.

Figure 4 presents the results of the meta-analysis of studies on intermediate resistance to ciprofloxacin with the proportions of mutations in the *gyr*A gene. The results of the random-effects model indicate that 91% (CI 78–97%) of the isolates had mutations in the *gyr*A gene.

**Figure 5:**
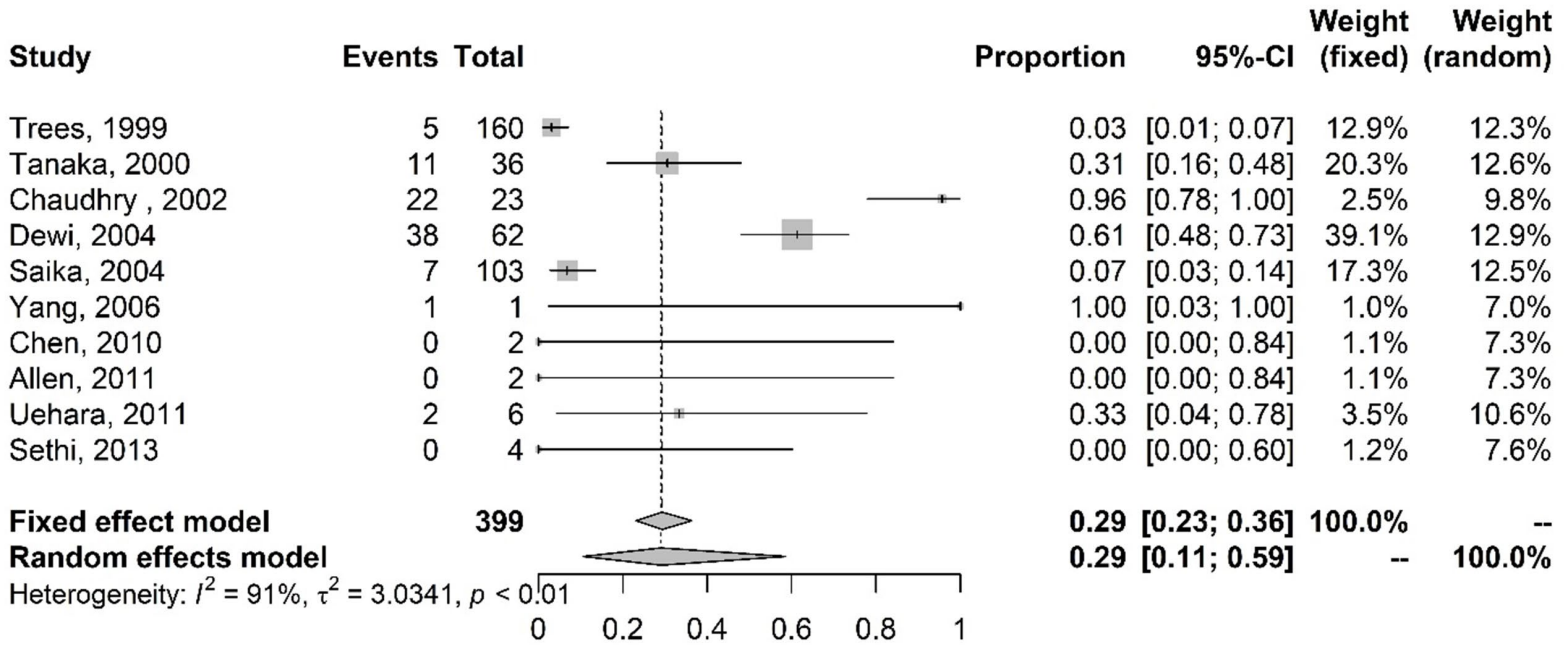
Meta-analysis of the proportion of gene mutations in isolates with intermediate resistance to ciprofloxacin (*par*C gene). The results are presented in forest plots. In its first column (“study”) are the citations of the selected studies. The second column (“events”) contains the number of isolates with mutations in the evaluated gene, while the third column (“total”) shows the total number of samples analyzed in that study. The fourth column presents the proportion estimate followed by their confidence intervals. The sixth and seventh columns present, respectively, the weights for the fixed and random models of the meta-analysis.

The meta-analysis results of studies on intermediate resistance to ciprofloxacin with the proportions of mutations in the *par*C gene are shown in Figure 5. Random-effects model results indicate that 29% (CI 11–59%) of the isolates had mutations in the *par*C gene.

### 3.8 Meta-analysis of studies evaluating tetracycline resistance

**Figure 6:**
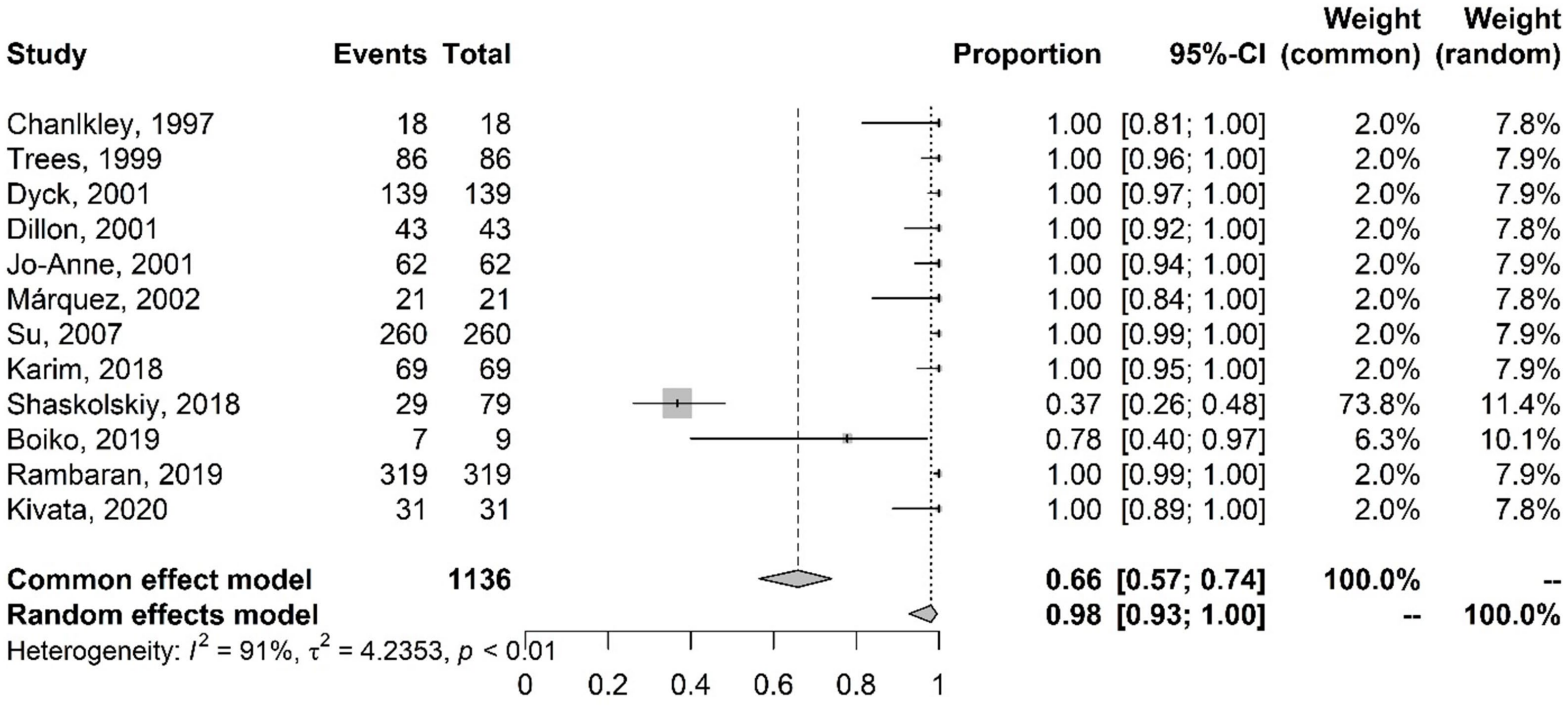
Meta-analysis of the proportion of gene mutations in tetracycline-resistant isolates and the presence of the *tet*M gene. The results are presented in forest plots. In its first column (“study”) are the citations of the selected studies. The second column (“events”) contains the number of isolates with mutations in the evaluated gene, while the third column (“total”) shows the total number of samples analyzed in that study. The fourth column presents the proportion estimate followed by their confidence intervals. The sixth and seventh columns present, respectively, the weights for the fixed and random models of the meta-analysis.

The presence of the plasmid containing the *tet*M gene and tetracycline resistance was also evaluated. Figure 6 presents the results of the meta-analysis of studies on the antibiotic tetracycline with the proportions of plasmids containing the *tet*M gene. Little variability and random error are observed. The random-effects model results indicate that 98% of the analyzed isolates had the *tet*M gene.

## 4 Discussion

The present study estimated the frequency of mutations in the genes of *N. gonorrhoeae* isolates conferring resistance to the antibiotics penicillin, cefixime, ceftriaxone, azithromycin and ciprofloxacin, and the presence of the *tet*M gene conferring resistance to tetracycline. As shown by the results obtained in this study, researchers in several countries have studied mechanisms of resistance of *N. gonorrhoeae* to antibiotics. It is evident that *N. gonorrhoeae* has developed or acquired resistance to several classes of antibiotics. Here, it was possible to determine the existence of resistance and/or reduced susceptibility to penicillin, ceftriaxone, cefixime, tetracycline, azithromycin and ciprofloxacin and show that mutations in genes encoding target proteins of these antibiotics are present in more than 50% of the isolates. Cases with elevated MICs for third-generation cephalosporins have been reported worldwide. These isolates are of concern, as these antibiotics are the last option for treating gonorrhoea.

**Figure 7:**
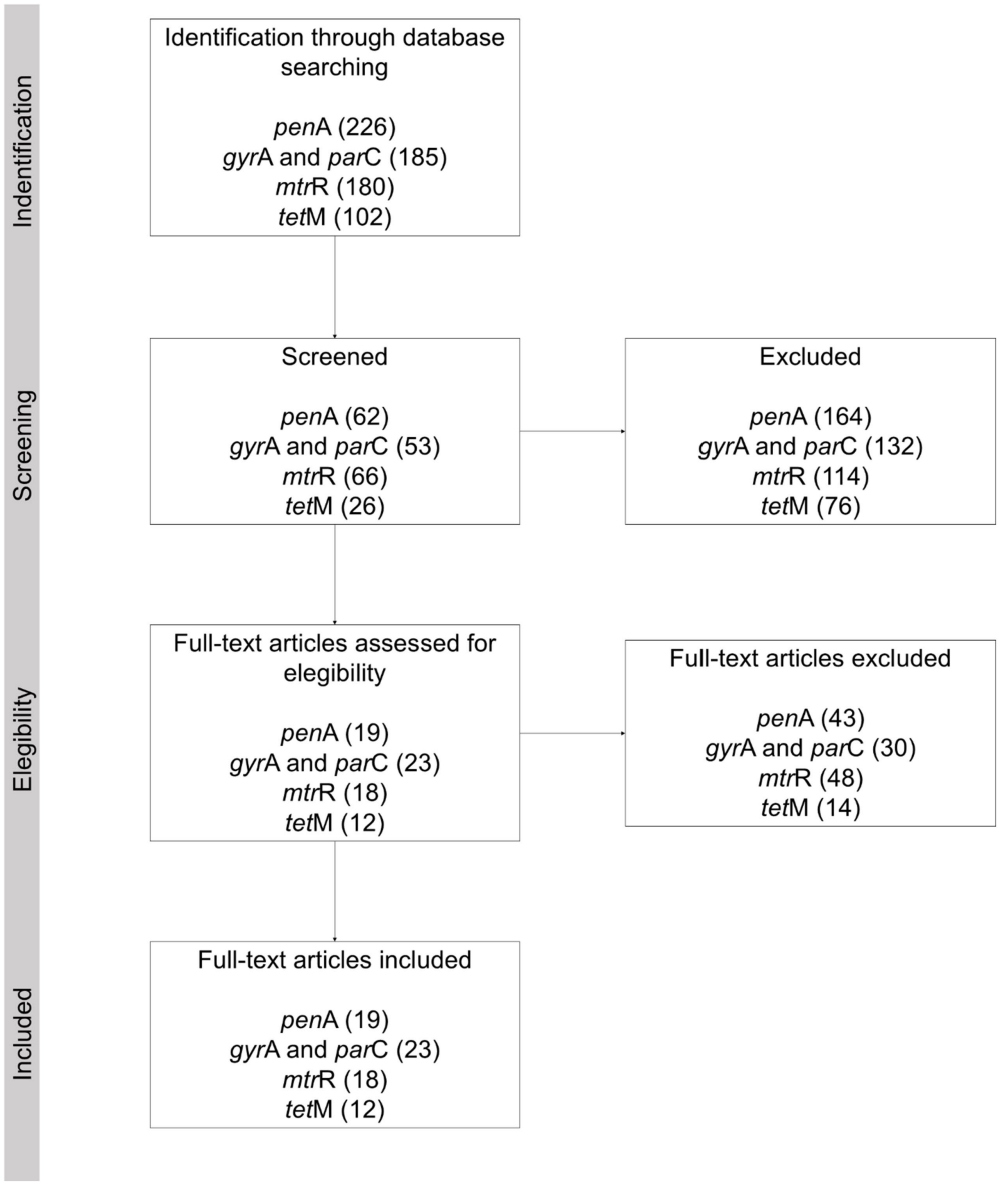
PRISMA flowchart.

The results of meta-analyses of the *pen*A and *mtr*R genes revealed that there is a relationship between reduced susceptibility and/or resistance to penicillin, ceftriaxone and cefixime and mutations in these genes in different countries over time. In addition, the meta-analyses of studies on the *gyr*A and *par*C genes showed that mutations in these genes are involved in ciprofloxacin resistance. Moreover, the presence of a plasmid containing the *tet*M gene is related to tetracycline resistance, as shown by the meta-analysis of studies on this gene. The results also showed that mutations in the *mtr*R gene are involved in the development of resistance to cefixime, ceftriaxone and azithromycin. The relationship between the presence of mutations and antibiotic resistance was supported by the presence of mutations in more than 50% of isolates with resistance, intermediate resistance or reduced susceptibility.

The studies also evaluated the main mutations found (Supplementary materials). The most common mutations in *pen*A contribute to resistance to *β*-lactams. The A501V, A501T and G545S substitutions may increase the MICs across the spectrum of cephalosporins (Unemo and Golparian, 2019). Mosaic-like structures in PBP2 were found in several isolates (Table 1 and 2, column 3). Meanwhile, Takahata et al. (2006) found that the presence of mosaicism in PBP2 was strongly associated with reduced susceptibility to cefixime and other cephalosporins. Commensal *Neisseria* are reservoirs of resistance genes and are linked to the increase in *N. gonorrhoeae* resistant isolates. Takahata et al. (2006) proposed that horizontal transfer of *pen*A genes among *Neisseria* resulted in allelic mosaicism in *N. gonorrhoeae* and *N. meningitidis*. However, some substitutions such as G545S are the result of selective pressure by antibiotics.

Mutations in *mtr*R, such as the deletion of alanine (A) in the 13 bp inverted repeat sequence in the promoter region and more common substitutions such as G45D in the coding region, were shown to cause the overexpression of MtrCDE efflux pumps and the increase in efflux. These were in turn revealed to be related to resistance to penicillin and azithromycin and reduced susceptibility to cefixime and ceftriaxone (Costa-Lourenço et al., 2017). The efflux pump system is one of the essential factors behind multi-drug resistance (Tanaka et al., 2004). The presence of other mutations such as in *mtr*R may be necessary for the high MIC level of ceftriaxone (Jamaludin et al., 2019).

Meta-analysis results of the *gyr*A and *par*C genes showed that there is a higher proportion of mutations in *gyr*A than in *par*C, mainly in isolates with intermediate resistance. Costa-Lourenço et al. (2017) suggested that mutations in *gyr*A are more important for resistance to the quinolone ciprofloxacin as, in most cases, there is no mutation only in *par*C. In addition, Yang et al. (2006) suggested that mutations in *gyr*A determine the resistance in *N. gonorrhoeae*, while mutations in *par*C are related to a high level of resistance. The most common mutations in *gyr*A, such as S91F and D95N substitutions, decreased the binding of fluoroquinolones to the DNA gyrase subcomponent, while changes in *par*C such as D86N and S88P substitutions decreased the binding of fluoroquinolones to the topoisomerase IV subcomponents (Unemo and Shafer, 2014).

One of the characteristics of the studies that drew attention is the greater number of samples collected from male individuals in the studies that provide information on the sex of the subjects. Given that men are generally more symptomatic than women (Quillin and Seifert, 2018), the demand for care by men is greater, which explains the greater amount of information related to this group. Data on sex, sexual behavior and age may be relevant given that there is a higher incidence of gonorrhoea cases in particular populations, such as men who have sex with men, sex workers and young adults. Highlighting these data may be important to achieve a more complete analysis of the epidemiology of infection. However, few studies have reported this information. The incompleteness of the data makes it difficult to assess the quality of studies in the literature. The STROBE (Strengthening the reporting of observational studies in epidemiology) statement (STROBE, 2022) has recommendations that can improve the quality of epidemiological research reports.

Notably, there is an abundance of studies from Asia, such as China and Japan, but limited research from Latin America and Africa. The lack of Latin American and African epidemiological studies makes it difficult to monitor the infection and the advance of antibiotic resistance in these regions, while also affecting the clinical management of infections there.

The results also demonstrate that the relevant mutations are consistent over time and between different countries and regions. These epidemiological analyses are essential for the control of gonorrhoea, as measures to prevent the spread can be focused on the populations of interest. Studies on mutations and their connection with antibiotic resistance are essential for monitoring the infection.

Several countries reported lower effectiveness of azithromycin and ceftriaxone. Given that *N. gonorrhoeae* is naturally competent for transformation, as with other *Neisseria*, monitoring resistant isolates and adequate clinical management for gonorrhoea are essential. In addition, inadequate clinical management for other bacterial infections also accelerates the increase in resistance, favoring the transfer of resistance genes among bacteria. The emergence and spread of antibiotic-resistant *N. gonorrhoeae* may result in irreversible intractable gonorrhoea.

## 5 Conclusion

The development of resistance of gonorrhoea to various antibiotics has led to changes in treatment over the years. Genetic studies, which identify the mechanisms of resistance, and studies on pathology, evolution and epidemiology are necessary to establish adequate treatment for this disease. Moreover, such studies in combination with the monitoring of transmission and increased resistance may even contribute to the development of new antibiotics. Furthermore, until an effective treatment or vaccine for gonorrhoea is developed, activities to contain its spread and slow its rise of resistance are essential. These activities include prevention, early diagnosis, epidemiological monitoring, adequate treatment and awareness programs. The results of this study highlight the urgency of continuously monitoring these resistance genes. Putting these measures into practice will positively affect the quality of life of infected individuals and reduce the financial burden on healthcare systems.

## Data Availability

The datasets presented in this study can be found in online repositories. The names of the repository/repositories and accession number(s) can be found in the article/Supplementary material.
