# Supplementary material for: The frequency of mutations in the penA, mtrR, gyrA and parC genes of Neisseria gonorrhoeae, the presence of tetM gene and antibiotic resistance/susceptibility: a systematic review and meta-analyses
Source: Front Microbiol. 2025 Jan 22;15:1414330. doi: 10.3389/fmicb.2024.1414330 (PMC11808683; doi:10.3389/fmicb.2024.1414330)
Supplement: Supplementary file 1 [file Data_Sheet_1.pdf]

SUPPLEMENTARY MATERIAL -TABLES

Table 1. Characteristic of studies of isolates with reduced susceptibility to ceftriaxone and cefixime.

Author	Year of publication	Study period	Local	Number of isolates	Number of isolates screened	Antibiotics	Gene	M	W	MSM	MSW	SW	Men age	Women age	Anatomic site
[1]	2006	2002	Japan	58	58	Cefixme	*pon*A, *pen*A, *por*	NA	NA	NA	NA	NA	NA	NA	Urethra
[2]	2006	2000 - 2001	Japan	398	398	Ceftriaxone	*pen*A, *pon*A, *mtr*R	NA	NA	NA	NA	NA	NA	NA	Urethra
[3]	2011	2005 - 2008	China	230	230	Ceftriaxone	*pen*A, *mtr*R, *por*	230	0	NA	NA	NA	NA	NA	NA
[4]	2011	2008	Canada	149	149	Ceftriaxone, Cefixime,	*pen*A, *pon*A, *mtr*R, *por*	NA	NA	NA	NA	NA	NA	NA	NA
[5]	2013	2011	Vietnam	108	108	Ceftriaxone, Cefixime	*pen*A, *mtr*R, *por*B	85	19	NA	NA	NA	28.5	29	NA
[6]	2014	2011 e 2012	China	340	334	Cefixime	*pen*A, *mtr*R, *por*B, *pon*A	340	NA	NA	NA	NA	NA	NA	Urethra
[7]	2016	2011 - 2014	Italy	900	65	Cefixime	*pen*A, *mtr*R	NA	NA	NA	NA	NA	NA	NA	NA
[8]	2017	2014 - 2015	China	126	126	Cefixime, Ceftriaxone	*pen*A, *mtr*R	NA	NA	NA	NA	NA	NA	NA	Uretrha, cervice
[9]	2017	2015 - 2016	China	128	128	Ceftriaxone	*pen*A, *mtr*R, *pen*B	120	8	NA	NA	NA	32,8±10,1	34,8 +- 9,4	Urethra, endocervice
[10]	2017	2009 - 2013	Argentina	1987	1987	Ceftriaxone, Cefixime	*pen*A, *mtr*R	NA	NA	NA	NA	NA	NA	NA	NA
[11]	2018	2012 - 2015	New Zealand	28	28	Penicillin	*pen*A, *mtr*R, *pen*B, *pon*A	NA	NA	NA	NA	NA	NA	NA	NA
[12]	2019	2009 - 2017	Netherlands	348	348	Ceftriaxone	*pen*A	320	28	314	6	15	NA	NA	Urethra, rectum, cervice, pharynx
[13]	2019	2015 - 2017	Russia	522	522	Penicillin, Ceftriaxone	*pen*A	NA	NA	NA	NA	NA	NA	NA	NA

M= men. W= women. MSM = men who have sex with men. MSW = men who have sex with women. SW= sex workers. NA = not available.

Table 1. Characteristics of studies of isolates with reduced susceptibility to ceftriaxone and cefixime (cont.)

Author/Year of publication	Study period	Local	Antibiotics	Gene	Genes and mutations identification	Nucleotide sequencing	Antimicrobial susceptibility testing	MIC determinants	MIC breakpoints
[1]	2002	Japan	Cefixime	*pon*A, *pen*A, *por*	Isolated and amplified by PCR.	BigDye Terminator v1.1 cycle sequencing kit and 3730 DNA analyzer (Applied Biosystems).	MIC determined by an agar dilution technique.	Clinical and Laboratory Standards Institute guidelines (CLSI).	MIC ≥0.12 µg/ml (reduced susceptibility to cefixime).
[2]	2000 - 2001	Japan	Ceftriaxone	*pen*A, *pon*A, *mtr*R	Isolated and amplified by PCR.	DYEnamic ET Terminator Cycle Sequencing Kit (Amersham Biosciences, Piscataway, NJ) and ABI PRISM 3100 Genetic Analyzer (Applied Biosystems, Foster City, CA).	MIC determined by an agar dilution technique.	National Committee for Clinical Laboratory Standards (NCCLS).	MIC = 0.5µg/ml (reduced susceptibility to ceftriaxone).
[3]	2005-2008	China	Ceftriaxone	*pen*A, *mtr*R, *por*	Isolated and amplified by PCR.	Applied Biosystems 3730x1 DNA Analyzer.	MIC determined by an agar dilution technique.	Clinical and Laboratory Standards Institute guidelines (CLSI).	MIC = 0.125–0.25 mg/L (reduced susceptibility to ceftriaxone) MIC = 0.004–0.016 mg/L (susceptible to ceftriaxone)
[4]	2008	Canada	Ceftriaxone, Cefixime, Penicillin	*pen*A, *pon*A, *mtr*R, *por*	Isolated and amplified by PCR.	BigDye terminator methodology in a model 3130*xl* DNA sequence analyzer (Applied Biosystem/Perkin-Elmer, Foster City, CA).	MIC determined by an agar dilution technique.	Clinical and Laboratory Standards Institute guidelines (CLSI).	MIC = 0.125 to 0.25 µg/ml (reduced susceptibility to cefixime) MIC = 0.032 to 0.125 µg/ml (reduced

									susceptibility to ceftriaxone)
[5]	2011	Vietnam	Ceftriaxone, Cefixime	*pen*A, *mtr*R. *por*B	Isolated and amplified by PCR.	BigDye Terminator v3.1 Cycle Sequencing Kit (Applied Biosystems, Foster City, CA, USA) on a GeneAmp 2720 Thermal Cycler (Applied Biosystems).	MIC determined by Etest method (bioMerieux AB, Solna, Sweden).	The European Committee on Antimicrobial Susceptibility Testing (EUCAST).	MIC ≥ 0.125 mg/L (reduced susceptibility to ceftriaxone and cefixime)
[6]	2011-2012	China	Ceftriaxone	*pen*A, *mtr*R. *por*B. *pon*A	Isolated and amplified by PCR.	Applied Biosystems 3730XL DNA automatic sequencer.	MIC determined by an agar dilution technique.	Clinical and Laboratory Standards Institute guidelines (CLSI).	MIC ≥0.125 mg/L (reduced susceptibility to ceftriaxone).
[7]	2011-2014	Italy	Cefixime	*pen*A, *mtr*R	Isolated and amplified by PCR.	Automatic sequencer (model 3730xl; Applied Biosystems, Weiterstadt, Germany).	MIC determined by Etest (bioMe´rieux) and MIC TEST STRIP method (Liofilchem Diagnostici).	The European Committee on Antimicrobial Susceptibility Testing (EUCAST).	MIC >0.064–0.125 mg/L (reduced susceptibility to cefixime).
[8]	2014-2015	China	Cefixime, Ceftriaxone	*pen*A, *mtr*R	Isolated and amplified by PCR.	Applied Biosystems 3730XL DNA automatic sequencer.	MIC determined by an agar dilution technique.	Clinical and Laboratory Standards Institute guidelines (CLSI).	MIC ≥ 0.125 mg/L (reduced susceptibility to ceftriaxone) MIC ≥ 0. 25 mg/L (reduced susceptibility to cefixime).
[9]	2015-2016	China	Ceftriaxone	*pen*A, *mtr*R. *pen*B	Isolated and amplified by PCR.	NA	MIC determined by an agar dilution technique.	The European Committee on Antimicrobial Susceptibility	MIC ≥ 0.125 mg/L

								Testing (EUCAST).	(reduced susceptibility to ceftriaxone).
[10]	2009-2013	Argentina	Ceftriaxone, Cefixime	*pen*A, *mtr*R	Isolated and amplified by PCR.	BigDye terminator v3.1 cycle sequencing kits (Applied Biosystems, Foster City, Calif) on an ABI 3500 Genetic Analyzer (Applied Biosystems).	MIC determined by an agar dilution technique.	Clinical and Laboratory Standards Institute guidelines (CLSI).	MIC 0.06–0.25 mg/L (reduced susceptibility to ceftriaxone) MIC 0.125–0.25 mg/L (reduced susceptibility to cefixime).
[11]	2012-2015	New Zealand	Ceftriaxone	*pen*A, *mtr*R, *pen*B, *pon*A	Isolated and amplified by PCR.	NA	MIC determined by MIC test strips.	Institute of Environmental Science and Research Limited (ESR), Porirua, New Zealand, and the Australian Gonococcal Surveillance Programme (AGSP).	MIC = 0.06 to 0.125 mg/L (reduced susceptibility to ceftriaxone)
[12]	2009-2017	Netherlands	Ceftriaxone	*pen*A	Isolated and amplified by PCR.	BigDyeTerminator, sequencing buffer and sequenced using an ABI 3130 automated sequencer.	MIC determined by Etest method (bioMérieux, France).	Clinical and Laboratory Standards Institute guidelines (CLSI) and The European Committee on Antimicrobial Susceptibility Testing (EUCAST).	MIC ≥0.064 mg/L (reduced susceptibility to ceftriaxone).

[13]	2015-2017	Russia	Ceftriaxone	*pen*A	Isolated and amplified by PCR.	3730xl Genetic Analyzer (Applied Biosystems, USA).	MIC determined by an agar dilution technique.	The European Committee on Antimicrobial Susceptibility Testing (EUCAST).	MIC 0.25 mg/L and 0.125 mg/L (reduced susceptibility to ceftriaxone).

MIC = minimum inhibitory concentration. NA = not available.

Table 2. Characteristics of the studies of isolates resistant to penicillin, ceftriaxone, and cefixime.

Author	Year of publication	Study period	Local	Number of isolates	Number of isolates screened	Antibiotics	Gene	M	W	MSM	MSW	SW	Men age	Women age	Anatomic site
[14]	2006	NA	Russia	31	31	Penicillin	*pen*A, *pon*A, *pen*B	NA	NA	NA	NA	NA	NA	NA	NA
[4]	2011	2008	Canada	149	149	Ceftriaxone, Cefixime, Penicillin	*pen*A, *pon*A, *mtr*R, *por*	NA	NA	NA	NA	NA	NA	NA	NA
[5]	2013	2011	Vietnam	108	108	Ceftriaxone, Cefixime	*pen*A, *mtr*R, *por*	85	19	NA	NA	NA	28.5	29	NA
[15]	2014	2007- 2012	China	278	15	Ceftriaxone	*pen*A, *mtr*R, *pen*B	232	46	NA	NA	NA	NA	NA	Urethra, endocervice
[16]	2015	2011 - 2013	Korea	210	210	Ceftriaxone, Cefixime	*pen*A	136	47	NA	NA	27	NA	NA	NA
[17]	2016	2010 - 2014	France	6340	6340	Ceftriaxone	*pen*A, *mtr*R, *pen*B	NA	NA	NA	NA	NA	NA	NA	NA
[7]	2016	2011 - 2014	Italy	900	65	Cefixime	*pen*A, *mtr*R	NA	NA	NA	NA	NA	NA	NA	NA
[9]	2017	2015 - 2016	China	128	128	Ceftriaxone	*pen*A, *mtr*R, *pen*B	120	8	NA	NA	NA	32,8 ± 10,1	34,8 ± 9,4	Urethra, endocervice
[18]	2018	2003 - 2008	Canada	146	146	Penicillin	*pen*A, *mtr*R, *pen*B	NA	NA	NA	NA	NA	NA	NA	NA
[11]	2018	2012 - 2015	New Zaeland	28	28	Penicillin	*pen*A, *mtr*R, *pen*B, *pon*A	NA	NA	NA	NA	NA	NA	NA	NA
[19]	2019	2015 - 2017	China	379	379	Ceftriaxone	*pen*A	NA	NA	NA	NA	NA	NA	NA	NA
[20]	2019	2013 - 2015	Portugal	30	30	Penicillin, Cefixime	*pen*A, *mtr*R	30	0	30	0	NA	NA	NA	Urethra, anus
[21]	2020	2013 - 2018	Kenya	36	36	Penicillin	*pen*A, *pen*B, *mtr*R, *pon*A	NA	NA	NA	NA	NA	NA	NA	Urethra, endocevice

M= men. W= women. MSM = men who have sex with men. MSW = men who have sex with women. SW= sex workers. NA = not available.

Table 2. Characteristics of the studies of isolates resistant to penicillin, ceftriaxone and cefixime.

Author/Year of publication	Study period	Local	Antibiotics	Gene	Genes and mutations identification	Nucleotide sequencing	Antimicrobial susceptibility testing	MIC determinants	MIC breakpoints
[14].	NA	Russia	Penicillin	*pen*A, *pon*A, *pen*B	NA	Sequenced by mass spectrometry.	MIC determined by an agar dilution technique.	National Committee for Clinical Laboratory Standards (NCCLS).	MIC ≥ 0.25 μg/ml (resistance to penicillin).
[4]	2008	Canada	Penicillin	*pen*A, *pon*A, *mtr*R. *por*	Isolated and amplified by PCR.	BigDye terminator methodology in a model 3130*xl* DNA sequence analyzer (Applied Biosystem/Perkin-Elmer, Foster City, CA).	MIC determined by an agar dilution technique.	Clinical and Laboratory Standards Institute guidelines (CLSI).	MIC ≥ 2 μg/ml (resistance to penicillin).
[5]	2011	Vietnam	Ceftriaxone, Cefixime	*pen*A, *mtr*R, *por*	Isolated and amplified by PCR.	BigDye Terminator v3.1 Cycle Sequencing Kit (Applied Biosystems, Foster City, CA, USA) on a GeneAmp 2720 Thermal Cycler (Applied Biosystems).	MIC determined by Etest method (bioMerieux AB, Solna, Sweden).	The European Committee on Antimicrobial Susceptibility Testing (EUCAST).	MIC > 1.0 mg/l (resistance to penicillin) MIC >0.125 mg/l (resistance to ceftriaxone) MIC > 0.125 mg/l (resistance to cefixime).
[15]	2007-2012	China	Ceftriaxone	*pen*A, *mtr*R, *pen*B	Isolated and amplified by PCR.	NA	MIC determined by an agar dilution technique.	The European Committee on Antimicrobial Susceptibility Testing (EUCAST).	MIC >125mg/l (resistance to ceftriaxone).
[16]	2011-2013	Korea	Ceftriaxone, Cefixime	*pen*A	Isolated and amplified by PCR.	NA	MIC determined by an agar	The European Committee on	MIC > 0.12 mg/l (resistance to cefixime)

							dilution technique.	Antimicrobial Susceptibility Testing (EUCAST).	MIC >0.12mg/l (resistance to ceftriaxone).
[17]	2010-2014	France	Ceftriaxone	*pen*A, *mtr*R. *pen*B	DNA amplification was performed with the GS Junior Lib-L library.	Sequencing was carried out using the GS Junior XL + kit in the 454-GS Junior instrument.	MIC determined by Etest method (bioMérieux, France).	The European Committee on Antimicrobial Susceptibility Testing (EUCAST).	MIC > 0.125mg/l (resistance to ceftriaxone).
[7]	2011-2014	Italy	Cefixime	*pen*A, *mtr*R	Isolated and amplified by PCR.	Automatic sequencer (model 3730xl; Applied Biosystems, Weiterstadt, Germany).	MIC determined by Etest (bioMe´rieux) and MIC TEST STRIP method (Liofilchem Diagnostici).	The European Committee on Antimicrobial Susceptibility Testing (EUCAST).	MIC >0.125mg/l (resistance to cefixime).
[9]	2015-2016	China	Ceftriaxone	*pen*A, *mtr*R, *pen*B	Isolated and amplified by PCR.	NA	MIC determined by an agar dilution technique.	The European Committee on Antimicrobial Susceptibility Testing (EUCAST).	MIC >0.125mg/l (resistance to ceftriaxone).
[18]	2003-2008	Canada	Penicillin	*pen*A, *mtr*R, *pen*B	Isolated and amplified by PCR.	Applied Biosystems 3730*xl* DNA Analyzer instrument (Plant Biotechnology Institute, National Research Council of Canada, Saskatoon, SK, Canada).	MIC determined by an agar dilution technique.	Clinical and Laboratory Standards Institute guidelines (CLSI).	MIC = 2–4 mg/L (resistance to penicillin).
[11]	2012-2015	New Zaeland	Penicillin	*penA, mtrR, penB, ponA*	Isolated and amplified by PCR.	NA	MIC determined by MIC test strips.	The European Committee on Antimicrobial Susceptibility	NA

								Testing (EUCAST).	
[19]	2015-2017	China	Ceftriaxone	*pen*A	Isolated and amplified by PCR.	NA	MIC determined by an agar dilution technique.	The European Committee on Antimicrobial Susceptibility Testing (EUCAST).	MIC >0.125mg/l (resistance to ceftriaxone).
[20]	2013-2015	Portugal	Penicilin, Cefixime	*pen*A, *mtr*R	Isolated and amplified by PCR.	NA	MIC determined by E-test method.	The European Committee on Antimicrobial Susceptibility Testing (EUCAST).	NA
[21]	2013-2018	Kenya	Penicillin	*pen*A, *pen*B, *mtr*R, *pon*A	Whole-genome sequencing.	Illumina Nextera XT kit (Illumina Inc. San Diego, CA, USA) Sequence reads were generated on Illumina MiSeq platform (Illumina, San Diego, CA, USA) using a paired-end 2 × 300 bp protocol.	MIC determined by E-test method (Biomerieux).	The European Committee on Antimicrobial Susceptibility Testing (EUCAST).	MIC >1mg/l (resistance to penicillin).

MIC = minimum inhibitory concentration. NA = not available.

Table 3. Characteristics of studies on the *mtr*R gene of isolates with reduced susceptibility.

Author	Year of publication	Study period	Local	Number of isolates	Number of isolates analisados	Antibiotics	Gene	M	W	MSM	MSW	SW	Men age	Women age	Anatomic site
[2]	2006	2000 a 2001	Japan	398	398	Ceftriaxone	*pen*A, *pon*A, *mtr*R	NA	NA	NA	NA	NA	NA	NA	Urethra
[3]	2011	2005 e 2008	China	230	230	Ceftriaxone	*pen*A, *mtr*R, *por*	230	0	NA	NA	NA	NA	NA	NA
[4]	2011	2008	Canada	149	149	Ceftriaxone, Cefixime, Penicillin	*pen*A, *pon*A, *mtr*R, *por*	NA	NA	NA	NA	NA	NA	NA	NA
[5]	2013	2011	Vietnam	108	108	Ceftriaxone, Cefixime	*pen*A, *mtr*R, *por*B	85	19	NA	NA	NA	28.5	29	NA
[8]	2017	2014 e 2015	China	126	126	Cefixime, Ceftriaxone,	*pen*A, *mtr*R	NA	NA	NA	NA	NA	NA	NA	Urethra, cervice
[9]	2017	2015 - 2016	China	128	128	Ceftriaxone	*pen*A, *mtr*R, *pen*B	120	8	NA	NA	NA	32,8 ± 10,1	34,8 ± 9,4	Urethra endocervice
[10]	2017	2009 - 2013	Argentina	1987	1987	Ceftriaxone, Cefixime	*pen*A, *mtr*R	NA	NA	NA	NA	NA	NA	NA	NA
[17]	2016	2011 - 2014	Italy	900	65	Cefixime	*pen*A, *mtr*R	NA	NA	NA	NA	NA	NA	NA	NA
[11]	2018	2012 - 2015	New Zaeland	28	28	Penicillin	*pen*A, *mtr*R, *pen*B, *pon*A	NA	NA	NA	NA	NA	NA	NA	NA
[13]	2019	2015-2017	Russia	522	522	Penicillin, Ceftriaxone	*pena* mtrR	NA	NA	NA	NA	NA	NA	NA	NA

M= men. W= women. MSM = men who have sex with men. MSW = men who have sex with women. SW= sex workers. NA = not available.

Table 3. Characteristics of studies on the *mtr*R gene of isolates with reduced susceptibility.

Author/Year of publication	Study period	Local	Antibiotics	Gene	Genes and mutations identification	Nucleotide sequencing	Antimicrobial susceptibility testing	MIC determinants	MIC breakpoints
[2]	2000 - 2001	Japan	Ceftriaxone	*pen*A, *pon*A, *mtr*R	Isolated and amplified by PCR.	DYEnamic ET Terminator Cycle Sequencing Kit (Amersham Biosciences, Piscataway, NJ) and ABI PRISM 3100 Genetic Analyzer (Applied Biosystems, Foster City, CA).	MIC determined by an agar dilution technique.	National Committee for Clinical Laboratory Standards (NCCLS).	MIC = 0.5µg/ml (reduced susceptibility to ceftriaxone).
[3]	2005-2008	China	Ceftriaxone	*pen*A, *mtr*R, *por*	Isolated and amplified by PCR.	Applied Biosystems 3730x1 DNA Analyzer.	MIC determined by an agar dilution technique.	Clinical and Laboratory Standards Institute guidelines (CLSI).	MIC = 0.125–0.25 mg/L (reduced susceptibility to ceftriaxone) MIC = 0.004–0.016 mg/L (susceptible to ceftriaxone).
[4]	2008	Canada	Ceftriaxone, Cefixime, Penicillin	*pen*A, *pon*A, *mtr*R, *por*	Isolated and amplified by PCR.	BigDye terminator methodology in a model 3130*xl* DNA sequence analyzer (Applied Biosystem/Perkin-Elmer, Foster City, CA).	MIC determined by an agar dilution technique.	Clinical and Laboratory Standards Institute guidelines (CLSI).	MIC = 0.125 to 0.25 µg/ml (reduced susceptibility to cefixime) MIC = 0.032 to 0.125 µg/ml (reduced susceptibility to ceftriaxone).
[5]	2011	Vietnam	Ceftriaxone, Cefixime	*pen*A, *mtr*R. *por*B	Isolated and amplified by PCR.	BigDye Terminator v3.1 Cycle Sequencing Kit (Applied Biosystems, Foster City, CA,	MIC determined by Etest method (bioMerieux AB, Solna, Sweden).	The European Committee on Antimicrobial Susceptibility Testing (EUCAST).	MIC ≥ 0.125 mg/L (reduced susceptibility to ceftriaxone and cefixime).

						USA) on a GeneAmp 2720 Thermal Cycler (Applied Biosystems).			
[8]	2014 - 2015	China	Cefixime, Ceftriaxone	*pen*A, *mtr*R	Isolated and amplified by PCR.	Applied Biosystems 3730XL DNA automatic sequencer.	MIC determined by an agar dilution technique.	Clinical and Laboratory Standards Institute guidelines (CLSI).	MIC ≥ 0.125 mg/L (reduced susceptibility to ceftriaxone) MIC ≥ 0. 25 mg/L (reduced susceptibility to cefixime).
[9]	2015 - 2016	China	Ceftriaxone	*pen*A, *mtr*R. *pen*B	Isolated and amplified by PCR.	NA	MIC determined by an agar dilution technique	The European Committee on Antimicrobial Susceptibility Testing (EUCAST).	MIC ≥ 0.125 mg/L (reduced susceptibility to ceftriaxone).
[7]	2011 - 2014	Italy	Cefixime	*pen*A, *mtr*R	Isolated and amplified by PCR.	Automatic sequencer (model 3730xl; Applied Biosystems, Weiterstadt, Germany).	MIC determined by Etest (bioMe´rieux) and MIC TEST STRIP method (Liofilchem Diagnostici).	The European Committee on Antimicrobial Susceptibility Testing (EUCAST).	MIC >0.064–0.125 mg/L (reduced susceptibility to cefixime).
[10]	2009 - 2013	Argenti na	Ceftriaxone, Cefixime	*pen*A, *mtr*R	Isolated and amplified by PCR.	BigDye terminator v3.1 cycle sequencing kits (Applied Biosystems, Foster City, Calif) on an ABI 3500 Genetic Analyzer (Applied Biosystems).	MIC determined by an agar dilution technique.	Clinical and Laboratory Standards Institute guidelines (CLSI).	MIC 0.06–0.25 mg/L (reduced susceptibility to ceftriaxone) MIC 0.125–0.25 mg/L (reduced susceptibility to cefixime).
[11]	2012 - 2015	New Zealan d	Ceftriaxone	*pen*A, *mtr*R, *pen*B, *pon*A	Isolated and amplified by PCR.	NA	MIC determined by MIC test strips.	Institute of Environmental Science and Research Limited	MIC = 0.06 to 0.125 mg/L

								(ESR), Porirua, New Zealand, and the Australian Gonococcal Surveillance Programme (AGSP).	(reduced susceptibility to ceftriaxone).
[13]	2015-2017	Russia	Ceftriaxone	*pen*A, *mtr*R	Isolated and amplified by PCR.	3730xl Genetic Analyzer (Applied Biosystems, USA).	MIC determined by an agar dilution technique.	The European Committee on Antimicrobial Susceptibility Testing (EUCAST).	MIC =0.25 mg/L and 0.125 mg/L (reduced susceptibility to ceftriaxone).

MIC = minimum inhibitory concentration. NA = not available.

Table 4. Characteristics of studies on the *mtr*R gene of resistant isolates.

Author	Year of publication	Study period	Local	Number of isolates	Number of isolates analisados	Antibiotics	Gene	M	W	MSM	MSW	SW	Men age	Women age	Anatomic site
[5]	2013	2011	Vietnam	108	108	Ceftriaxone, Cefixime	*pen*A, *mtr*R, *por*B	85	19	NA	NA	NA	28.5	29	NA
[22]	2014	2007 - 2012	China	278	278	Ceftriaxone	*mtr*R, *pen*A	232	46	NA	NA	NA	36.4	35,2	Urethra, cervice
[23]	2016	2013 - 2014	France	970	970	Azithromicin	*mtr*R	NA	NA	NA	NA	NA	NA	NA	NA
[18]	2018	2003 - 2008	Canada	146	146	Penicillin	*pen*A, *mtr*R, *pen*B	NA	NA	NA	NA	NA	NA	NA	NA
[9]	2017	2015 - 2016	China	128	128	Ceftriaxone	*pen*A, *mtr*R, *pen*B	120	8	NA	NA	NA	32.8 +- 10.1	34,8 ± 9,4	Urethra, endocervice
[24]	2019	2001 - 2018	Taiwan	598	598	Azithromicin	*mtr*R	NA	NA	NA	NA	NA	NA	NA	Urethra, vagina, pus, eye, blood, surgical wound, gastric juice, synovial fluid, Bartholin abscess
[25]	2019	2014 - 2017	China	55	55	Azithromicin	*mtr*R	52	3	NA	NA	NA	32	32	Urethra, NA
[7]	2016	2011 - 2014	Italy	900	65	Cefixime	*pen*A, *mtr*R	NA	NA	NA	NA	NA	NA	NA	NA
[11]	2018	2012 - 2015	New Zaeland	28	28	Penicillin	*pen*A, *mtr*R, *pen*B, *pon*A	NA	NA	NA	NA	NA	NA	NA	NA
[20]	2019	2013 - 2015	Portugal	30	30	Penicillin, Cefixime	*pen*A, *mtr*R	30	0	30	0	NA	NA	NA	Urethra, anus
[21]	2020	2013 - 2018	Kenya	36	36	Penicillin	*pen*A, *pen*B, *mtr*R, *pon*A, *bla*	NA	NA	NA	NA	NA	NA	NA	Urethra, endocevice
[26]	2020	NA	South Africa	51	27	Penicillin, azithromicin, tetracycline	*mtr*R	51	0	32	19	NA	27	NA	Urethra

M= men. W= women. MSM = men who have sex with men. MSW = men who have sex with women. SW= sex workers. NA = not available.

Table 4. Characteristics of studies on the *mtr*R gene of resistance isolates.

Author/Year of publication	Study period	Local	Antibiotics	Gene	Gene and mutations identification	Nucleotide sequencing	Antimicrobial susceptibility testing	MIC determinants	MIC breakpoints
[5]	2011	Vietnam	Ceftriaxone, Cefixime	*pen*A, *mtr*R, *por*	Isolated and amplified by PCR.	BigDye Terminator v3.1 Cycle Sequencing Kit (Applied Biosystems, Foster City, CA, USA) on a GeneAmp 2720 Thermal Cycler (Applied Biosystems).	MIC determined by Etest method (bioMerieux AB, Solna, Sweden).	The European Committee on Antimicrobial Susceptibility Testing (EUCAST).	MIC > 1.0 mg/l (resistance to penicillin) MIC >0.125 mg/l (resistance to ceftriaxone) MIC > 0.125 mg/l (resistance to cefixime).
[22]	2007-2012	China	Ceftriaxone	*pen*A, *mtr*R, *pen*B	Isolated and amplified by PCR.	NA	MIC determined by an agar dilution technique.	The European Committee on Antimicrobial Susceptibility Testing (EUCAST).	MIC >125mg/l (resistance to ceftriaxone).
[23]	2013-2014	France	Azithromycin	*mtr*R	Isolated and amplified by PCR.	Automated DNA sequence analysis (3100 capillary array, Applied Biosystems).	MIC determined by Etest method (i2A, France and bioMérieux).	The European Committee on Antimicrobial Susceptibility Testing (EUCAST).	MIC > 0.5mg/l (resistance to azithromycin).
[18]	2003-2008	Canada	Penicillin	*pen*A, *mtr*R, *pen*B	Isolated and amplified by PCR.	Applied Biosystems 3730*xl* DNA Analyzer instrument (Plant Biotechnology Institute, National Research Council of Canada, Saskatoon, SK, Canada).	MIC determined by an agar dilution technique.	Clinical and Laboratory Standards Institute guidelines (CLSI).	MIC 2–4 mg/L (resistance to penicillin).

[9]	2015-2016	China	Ceftriaxone	*pen*A, *mtr*R, *pen*B	Isolated and amplified by PCR.	NA	MIC determined by an agar dilution technique.	The European Committee on Antimicrobial Susceptibility Testing (EUCAST).	MIC >0.125mg/l (resistance to ceftriaxone).
[24]	2001-2018	Taiwan	Azithromycin	*mtr*R	Isolated and amplified by PCR.	NA	MIC determined by an agar dilution technique.	The European Committee on Antimicrobial Susceptibility Testing (EUCAST).	MIC ≥ 1µg/ml (resistance to azithromycin).
[25]	2014-2017	China	Azithromycin	*mtr*R	Isolated and amplified by PCR.	NA	MIC determined by E-test method (Wenzhou Kangtai Biological Technology Co., Ltd.).	Clinical and Laboratory Standards Institute (CLSI).	MIC ≥ 1µg/ml (azithromycin resistance).
[7]	2011-2014	Italy	Cefixime	*pen*A, *mtr*R	Isolated and amplified by PCR.	Automatic sequencer (model 3730xl; Applied Biosystems, Weiterstadt, Germany).	MIC determined by Etest (bioMe´rieux) and MIC TEST STRIP method (Liofilchem Diagnostici).	The European Committee on Antimicrobial Susceptibility Testing (EUCAST).	MIC >0.125mg/l (resistance to cefixime).
[11]	2012-2015	New Zaeland	Penicillin	*pen*A, *mtr*R, *pen*B, *pon*A	Isolated and amplified by PCR.	NA	MIC detemrined by MIC test strips.	The European Committee on Antimicrobial Susceptibility Testing (EUCAST).	NA
[20]	2013-2015	Portugal	Penicilin, Cefixime	*penA, mtrR*	Isolated and amplified by PCR.	NA	MIC determined by E-test method.	The European Committee on Antimicrobial Susceptibility Testing (EUCAST).	NA

[21]	2013-2018	Kenya	Penicillin	*penA, penB, mtrR, pon*A	Whole-genome sequencing.	Illumina Nextera XT kit (Illumina Inc. San Diego, CA, USA) Sequence reads were generated on Illumina MiSeq platform (Illumina, San Diego, CA, USA) using a paired-end 2 × 300 bp protocol.	MIC determined by E-test method (Biomerieux).	The European Committee on Antimicrobial Susceptibility Testing (EUCAST).	MIC >1mg/l (resistance to penicillin).
[26]	2018-2019	South Africa	Penicillin, azithromycin, tetracycline	*mtr*R	Whole-genome seguencing.	Nextera XT library preparation kit (Illumina, Eindhoven, The Netherlands). Paired-end 250-bp indexed reads were generated on the Illumina MiSeq.	MIC determined by E-test method (bioMérieux, France).	The European Committee on Antimicrobial Susceptibility Testing (EUCAST).	NA for penicillin and tetracycline. For azithromycin, No EUCAST resistance breakpoint exists. Epidemiological cutoff value of 1.0 mg/l was used for interpretation.

MIC = minimum inhibitory concentration. NA = not available.

Table 5. Characteristic of *gyr*A and *par*C studies.

Author	Year of publication	Study period	Local	Number of isolates	Number of isolates screened	Antibiotics	Gene	M	W	MSM	MSW	SW	Men age	Women age	Anatomic site
[27]	1999	1994 - 1996	Far East and US	234	234	Ciprofloxacin	*gyrA, parC*	NA	NA	NA	NA	107	NA	NA	NA
[28]	2000	1991 - 1998	Netherlands	2352	2352	Ciprofloxacin	*gyrA, parC*	NA	NA	NA	NA	NA	NA	NA	NA
[29]	2000	1993 - 1997	Japan	85	85	Ciprofloxacin	*gyrA, parC*	NA	NA	NA	NA	NA	NA	NA	NA
[30]	2000	1993 - 1998	Japan	502	502	Ciprofloxacin	*gyrA, parC*	502	0	NA	NA	NA	NA	NA	NA
[31]	2002	2000 - 2001	India	63	63	Ciprofloxacin	*gyrA, parC*	NA	NA	NA	NA	NA	NA	NA	Urethra
[32]	2003	2000 - 2001	Spain	147	147	Ciprofloxacin	*gyrA, parC*	NA	NA	NA	NA	NA	NA	NA	NA
[33]	2004	1998 - 1999	Japan	332	332	Ciprofloxacin	*gyrA, parC*	200	132	NA	NA	NA	NA	NA	Urethra vagina
[34]	2004	1999 - 2003	Korea	817	817	Ciprofloxacin	*gyrA, parC*	NA	NA	NA	NA	NA	NA	NA	NA
[35]	2004	2002	Russia	32	32	Ciprofloxacin	*gyrA, parC*	NA	NA	NA	NA	NA	NA	NA	NA
[36]	2004	1999 - 2001	Japan	131	131	Ciprofloxacin	*gyrA, parC*	131	0	NA	NA	NA	NA	NA	NA
[37]	2004	1999 - 2002	France	104	104	Ciprofloxacin	*gyrA, parC*	NA	NA	NA	NA	NA	NA	NA	NA
[38]	2004	2000 -2001	Israel	80	80	Ciprofloxacin	*gyrA, parC*	53	27	53	NA	27	NA	NA	pharynx,
[39]	2006	2004 - 2005	China	159	103	Ciprofloxacin	*gyrA, parC*	159	NA	NA	NA	NA	NA	NA	NA
[40]	2006	2003	China	95	54	Ciprofloxacin	*gyrA, parC*	78	17		NA	NA	33-96	33-96	NA
[41]	2008	2004 - 2005	Russia	464	464	Ciprofloxacin	*gyrA, parC*	NA	NA	NA	NA	NA	NA	NA	NA
[42]	2010	1999 - 2004	Taiwan	90	45	Ciprofloxacin	*gyrA, parC*	NA	NA	NA	NA	NA	NA	NA	NA
[4]	2011	2008	Canada	149	149	Ciprofloxacin	*gyrA, parC*	NA	NA	NA	NA	NA	NA	NA	NA
[43]	2011	2005 - 2010	Brazil	125	125	Ciprofloxacin	*gyrA, parC*	NA	NA	NA	NA	NA	NA	NA	Urethra, vagina,
[44]	2012	2007 - 2009	India	64	64	Ciprofloxacin	*gyrA, parC*	49	15	2	NA	7	NA	NA	NA
[45]	2013	2007 - 2010	India,Pakistan,Buthan	65	65	Ciprofloxacin	*gyrA, parC*	60	5	NA	NA	NA	NA	NA	NA
[20]	2019	2013 - 2015	Portugal	30	30	Ciprofloxacin	*gyrA, parC*	30	NA	30	NA	NA	NA	NA	anus, Urethra
[46]	2019	NA	Kenya	84	22	Ciprofloxacin	*gyrA, parC*	73	11	NA	NA	NA	NA	NA	NA
[47]	2019	2013 - 2018	Ukraine	150	150	Ciprofloxacin	*gyrA, parC*	130	20	2	NA	NA	29,2	28	NA

M= men. W= women. MSM = men who have sex with men. MSW = men who have sex with women. SW= sex workers. NA = not available.

Table 5. Characteristics of *gyr*A and *par*C studies.

Author/ year of publication	Study period	Local	Antibiotics	Gene	Gene and mutations analysis	Nucleotide sequencing	Antimicrobial susceptibility testing	MIC determinants	MIC breakpoints
[27]	1994-1996	Far East and US	Ciprofloxacin	*gyr*A, *par*C	Isolated and amplified by PCR.	NA	MIC determined by an agar dilution technique.	National Committee for Clinical Laboratory Standards (NCCLS)	MIC ≥ 1.0mg/l (resistance to ciprofloxacin) MIC 0.06–0.5 mg/l (intermediate resistance to ciprofloxacin) NA (susceptible to ciprofloxacin)
[28]	1991-1998	Netherlands	Ciprofloxacin	*gyr*A, *par*C	Isolated and amplified by PCR.	ABI PRISM Big Dye cycle sequencing ready reaction kit on a 377 DNA sequencer (Perkin-Elmer, Applied Biosystems, Foster City, Calif.).	MIC determined by an agar dilution technique.	NA	MIC ≥ 1µg/ml (resistance to ciprofloxacin) MIC 0.12 to 0.5 µg/ml (intermediate resistance to ciprofloxacin) NA (susceptible to ciprofloxacin)
[29]	1993-1997	Japan	Ciprofloxacin	*gyr*A, *par*C	Isolated and amplified by PCR.	Dideoxy-chain termination method with the Taq DyeDeoxy Terminator Cycle Sequencing Kit and a model 373A autosequencer (ABI).	MIC determined by an agar dilution technique.	National Committee for Clinical Laboratory Standards (NCCLS)	MIC ≥ 1 µg/ml (resistance to ciprofloxacin) MIC 0.06–0.5 mg/l (intermediate resistance to ciprofloxacin) MIC ≤ 0.06 µg/ml (susceptible to ciprofloxacin)

[30]	1993-1998	Japan	Ciprofloxacin	*gyr*A, *par*C	Isolated and amplified by PCR.	*Taq* DyeDeoxy terminator cycle-sequencing kit and a Model 373A autosequencer (Applied Biosystems, Foster City, Calif.).	MIC determined by an agar dilution technique.	National Committee for Clinical Laboratory Standards (NCCLS)	MIC ≥1μg/ml (resistance to ciprofloxacin) MICs ≥0.125 μg/ml (decreased susceptibility to ciprofloxacin) MIC ≤0.06μg/ml (susceptible to ciprofloxacin)
[31]	2000-2001	India	Ciprofloxacing	*gyr*A, *par*C	Isolated and amplified by PCR.	*Taq* DyeDeoxy terminator cycle-sequencing kit and a Model 373A autosequencer (Applied Biosystems, Foster City, Calif.).	MIC determined by Etest method.	Knapp JS, Hale JA, Neal SW, et al. Proposed criteria for interpretation of susceptibilities of strains of Neisseria gonorrhoeae to ciprofloxacin, ofloxacin, enoxacin, lomefloxacin and norfloxacin. Antimicrob Agents Chemother 1995;39:2442–5.	MIC ≥1 μg/ml) (resistance to ciprofloxacin) MIC 0.06 μg/ml to 0.75 μg/ml (intermediate resistance to ciprofloxacin) MIC <0.06μg/ml (susceptible to ciprofloxacin)
[32]	2000-2001	Spain	Ciprofloxacin	*gyr*A, *par*C	Isolated and amplified by PCR.	NA	MIC determined by an agar dilution technique and confirmerd by E-test method.	NA	MIC ≤0.06 μg/mL (susceptible to ciprofloxacin) MIC 0.12 to 0.5 μg/mL (intermediate resistance to ciprofloxacin)

									MIC ≥1 μg/mL (resistance to ciprofloxacin)
[33]	1998-1999	Japan	Ciprofloxacin	*gyr*A, *par*C	Isolated and amplified by PCR.	373A DNA Sequencer (Applied Biosystems, Foster City, Calif., USA).	MIC determined by an agar dilution technique.	National Committee for Clinical Laboratory Standards (NCCLS)	MIC ≤ 0.06µg/ml (susceptible to ciprofloxacin) MIC 0.12–0.5µg/ml (intermediate resistance to ciprofloxacin) MIC ≥1µg/ml (resistance to ciprofloxacin)
[34]	1999-2003	Korea	Ciprofloxacin	*gyr*A, *par*C	Isolated and amplified by PCR.	Taq DyeDeoxy terminator cycle sequencing kit	MIC determined by an agar dilution technique.	National Committee for Clinical Laboratory Standards (NCCLS)	NA
[35]	2002	Russia	Ciprofloxacin	*gyr*A, *par*C	Isolated and amplified by PCR.	ABI Prism BigDye Terminator Cycle Sequencing Ready Reaction Kit and the ABI Prism 3100 Genetic Analyser (Applied Biosystems, USA; Hitachi, Japan).	MIC determined by an agar dilution technique.	National Committee for Clinical Laboratory Standards (NCCLS).	NA
[36]	1999-2001	Japan	Ciprofloxacin	*gyr*A, *par*C	Isolated and amplified by PCR.	BigDye Terminator Cycle Sequencing kit and an ABI PRISM 310 Genetic Analyzer	MIC determined by an agar dilution technique.	National Committee for Clinical Laboratory Standards (NCCLS).	MIC ≤0,06µg/ml (susceptible to ciprofloxacin) MIC 0.12 to 0.5µg/ml

						(Applied Biosystems).			(intermadiate resistance to ciprofloxacin) MIC ≥1µg/ml (resistance to ciprofloxacin).
[37]	1999-2002	France	Ciprofloxacin	*gyr*A, *par*C	Isolated and amplified by PCR.	The presence of mutations was determined when the enzymes failed to digest the amplified PCR products. The results obtained by restriction fragment analysis were confirmed by nucleotide sequencing of the PCR product.	MIC determined by an agar dilution technique.	National Committee for Clinical Laboratory Standards (NCCLS).	NA
[38]	2000-2001	Israel	Ciprofloxacin	*gyr*A, *par*C	Isolated and amplified by PCR.	BigDye Terminator Assay on a 3700 DNA Analyzer (Applied Biosystems).	MIC determined by Etest method (AB Biodisk, Solna, Sweden).	National Committee for Clinical Laboratory Standards (NCCLS).	MIC <0,125µg/ml (susceptible to ciprofloxacin) MIC ≥0,125µg/ml (intermediate resistance to ciprofloxacin) MIC ≥1µg/ml (resistance to ciprofloxacin).
[39]	2004-2005	China	Ciprofloxacin	*gyr*A, *par*C	Isolated and amplified by PCR.	NA	MIC determined by an agar dilution technique.	National Committee for Clinical Laboratory Standards (NCCLS).	NA

[40]	2003	China	Ciprofloxacin	*gyr*A, *par*C	Isolated and amplified by PCR.	ABI377 automatic sequencer (Applied Biosystems).	MIC determined by an agar dilution technique.	National Committee for Clinical Laboratory Standards (NCCLS).	NA
[41]	2004-2005	Russia	Ciprofloxacin	*gyr*A, *par*C	Isolated and amplified by PCR.	Detected by minisequencing reactions and modified Sanger method, using an ABI Prism BigDye Terminator cycle sequencing ready reaction kit and an ABI Prism 3100 genetic analyzer (Applied Biosystems; Hitachi, Japan).	MIC determined by an agar dilution technique.	Clinical and Laboratory Standards Institute (CLSI).	NA
[42]	1999-2004	Taiwan	Ciprofloxacin	*gyr*A, *par*C	Isolated and amplified by PCR.	NA	MIC determined by Etest (AB Biodisk, Solna, Sweden).	Clinical and Laboratory Standards Institute (CLSI).	MIC ≤0,06µg/ml (susceptible to ciprofloxacin) MIC 0.12 to 0.5µg/ml (intermadiate resistance to ciprofloxacin) MIC ≥1µg/ml (resistance to ciprofloxacin)
[4]	2008	Canada	Ciprofloxacin	*gyr*A, *par*C	Isolated and amplified by PCR.	BigDye terminator methodology	MIC determined by an agar dilution technique.	Clinical and Laboratory Standards	MIC ≤0,06µg/ml (susceptible to ciprofloxacin)

						in a model 3130*xl* DNA sequence analyzer (Applied Biosystem/Perkin-Elmer, Foster City, CA).		Institute (CLSI).	MIC 0.12 to 0.5µg/ml (intermediate resistance to ciprofloxacin) MIC ≥1µg/ml (resistance to ciprofloxacin).
[43]	2005-2010	Brazil	Ciprofloxacin	*gyr*A, *par*C	Isolated and amplified by PCR.	BigDye; Applied Biosystems, Foster City, CA on Applied Biosystems ABI Prism 3730 automated DNA sequencer.	MIC determined by Etest method (AB Biodisk. Solna, Sweden).	Clinical and Laboratory Standards Institute (CLSI).	MIC ≤0.06 µg/ml (susceptible to ciprofloxacin) MIC 0.125µg/ml to 0.75µg/ml (intermediate resistance to ciprofloxacin) MIC ≥ 1 µg/ml (resistance to ciprofloxacin).
[44]	2007-2009	India	Ciprofloxacin	*gyr*A, *par*C	Isolated and amplified by PCR.	BigDye; Applied Biosystems, Foster City, CA) on an ABI Prism 3100 Automated DNA Sequencer (Applied Biosystems).	Determined by Etest (AB BIODISK, Solna, Sweden).	Clinical and Laboratory Standards Institute (CLSI).	MIC ≥ 1 µg/ml (resistance to ciprofloxacin) MICs for susceptible and intermediate resistance was not available.
[45]	2007-2010	India, Pakistan, Buthan	Ciprofloxacin	*gyr*A, *par*C	Isolated and amplified by PCR.	BigDye Terminator v3.1 Cycle Sequencing Kit (Applied Biosystems, Foster City, CA, USA) on a GeneAmp 2720 Thermal Cycler (Applied Biosystems).	Determined by Etest.	The European Committee on Antimicrobial Susceptibility Testing (EUCAST).	MIC ≤0.03mg/l (susceptible to ciprofloxacin) MIC >0.06mg/l (resistance to ciprofloxacin).

[20]	2013-2015	Portugal	Ciprofloxacin	*gyr*A, *par*C	Isolated and amplified by PCR.	NA	Determined by Etest.	The European Committee on Antimicrobial Susceptibility Testing (EUCAST).	NA
[46]	NA	Kenya	Ciprofloxacin	*gyr*A, *par*C	Whole genomes were sequenced using Illumina platform.	Mutations in the GC Quinolone Resistant Determining Regions identified using Bioedit sequence alignment editor.	Determined by E-test (BioMerieux).	The European Committee on Antimicrobial Susceptibility Testing (EUCAST).	MIC ≤0.03mg/l (susceptible to ciprofloxacin) MIC >0.06mg/l (resistance to ciprofloxacin).
[47]	2013-2018	Ukraine	Ciprofloxacin	*gyr*A, *par*C	Whole genome sequencing using the Nextera XT DNA library preparation kit and the Illumina MiSeq Platform.	Whole genome sequencing using the Nextera XT DNA library preparation kit and the Illumina MiSeq Platform, and mutations identified *in silico* based on the *de novo* assembly.	Determined by Etest (bioMérieux, Marcy-l'Étoile, France).	The European Committee on Antimicrobial Susceptibility Testing (EUCAST).	NA

MIC = minimum inhibitory concentration. NA = not available.

Tabela 6. Characteristics of studies on the *tet*M gene.

Author	Year of publication	Study period	Local	Number of isolates	Number of isolates screened	Antibiotics	Gene	M	W	MSM	MSW	SW	Men age	Women age	Anatomic site
[48]	1997	1994 e 1995	South Africa	210	210	Tetracycline	*tet*M	NA	NA	NA	NA	NA	NA	NA	NA

[27]	1999	1994	Ohio	591	591	Tetracycline	*tet*M	NA	NA	NA	NA	NA	NA	NA	NA
[49]	2001	1998 a 1999	Benin	170	143	Tetracycline	*tet*M	0	143	0	0	143	NA	NA	vagina
[50]	2001	1994 - 1996	Guyana, Saint Vincent, Trinidad	1686	1686	Tetracycline	*tet*M	NA	NA	NA	NA	NA	NA	NA	NA
[51]	2001	1999	Manaus/Brazil	168	81	Tetracycline	*tet*M	139	29	NA	NA	NA	NA	NA	Urethra, cérvice
[52]	2002	1996 - 1999	Uruguay	181	181	Tetracycline	*tet*M	NA	NA	NA	NA	NA	NA	NA	NA
[53]	2007	1999 - 2006	China	1208	1208	Tetracycline	*tet*M	NA	NA	NA	NA	NA	NA	NA	NA
[54]	2018	2013 - 2015	Morocco	149	149	Tetracycline	*tet*M	0	149	0	0	NA	-	43,16	NA
[13]	2018	2015 - 2017	Russia	401	401	Tetracycline	*tet*M	322	79	NA	NA	NA	12 a 60	12 a 60	NA
[47]	2019	2013 - 2018	Ukraine	150	150	Tetracycline	*tet*M	NA	NA	NA	NA	NA	NA	NA	NA
[55]	2019	2013 - 2014	South Africa	319	319	Tetracycline	*tet*M	248	71	NA	NA	NA	NA	NA	Urethra, cérvice
[56]	2020	2013 - 2018	Kenya	36	36	Tetracycline	*tet*M	NA	NA	NA	NA	NA	NA	NA	Urethra, cérvice

M= men. W= women. MSM = men who have sex with men. MSW = men who have sexwith women. SW= sex workers. NA = not available.

Table 6. Characteristics of studies on the *tet*M gene.

Author/Year of publication	Study period	Local	Antibiotics	Gene	Gene identification	Antimicrobial susceptibility testing	MIC determinants	MIC breakpoints

[48]	1994-1995	South Africa	Tetracycline	*tet*M	Isolates was amplified by PCR to determine the presence of tetM.	MIC determined by an agar dilution technique.	NA	MIC ≥16mg/l (high-level tetracycline-resistance).
[27]	1994	Ohio	Tetracycline	*tet*M	Isolates was amplified by PCR to determine the presence of tetM.	MIC determined by an agar dilution technique.	National Committee for Clinical Laboratory Standards (NCCLS).	MIC ≥2.0µg/ml (resistance to tetracycline).
[49]	1998-1999	Benin	Tetracycline	*tet*M	NA	NA	NA	NA
[50].	1994-1996	Guyana, Saint Vincent, Trinidad	Tetracycline	*tet*M	Isolates was amplified by PCR to determine the presence of tetM.	MIC determined by an agar dilution technique.	National Committee for Clinical Laboratory Standards (NCCLS).	MIC ≥16µg/ml (resistance to tetracycline).
[51]	1999	Brazil	Tetracycline	*tet*M	NA	MIC determined by an agar dilution technique.	National Committee for Clinical Laboratory Standards (NCCLS).	MIC ≥16µg/ml (resistance to tetracycline).
[52].	1996-1999	Uruguay	Tetracycline	*tet*M	Isolates was amplified by PCR to determine the presence of tetM.	MIC determined by an agar dilution technique.	National Committee for Clinical Laboratory Standards (NCCLS).	MIC ≥16µg/ml (resistance to tetracycline).
[53]	1999-2006	China	Tetracycline	*tet*M	Isolates was amplified by PCR to determine the presence of tetM.	MIC determined by an agar dilution technique.	WHO WPR Resistance Surveillance Programme guidelines.	MIC ≥16mg/l (resistance to tetracycline).
[54]	2013-2015	Morocco	Tetracycline	*tet*M	Isolates was amplified by PCR to determine the presence of tetM.	NA	NA	NA

[13]	2015-2017	Russia	Tetracycline	*tet*M	Oligonucleotide low-density microarray was used to identify the presence of tetM gene.	MIC determined by an agar dilution technique.	The European Committee on Antimicrobial Susceptibility Testing (EUCAST).	MIC ≤ 0.5 mg/L (susceptible to tetracycline) MIC > 0.5 ≤ 1 mg/L (intermediate) MIC > 1 mg/L (resistance to tetracycline).
[47]	2013-2018	Uckaine	Tetracycline	*tet*M	Whole genome sequencing using the Nextera XT DNA library preparation kit and the Illumina MiSeq Platform.	MIC determined by Etest (bioMérieux, Marcy-l'Étoile, France).	The European Committee on Antimicrobial Susceptibility Testing (EUCAST).	NA
[55]	2013-2014	South Africa	Tetracycline	*tet*M	The tetM gene was amplified by PCR.	MIC determined by an agar dilution technique.	The European Committee on Antimicrobial Susceptibility Testing (EUCAST).	MIC >16mg/l.
[56]	2013-2018	Kenya	Tetracycline	*tet*M	Whole genome sequencing using Illumina Nextera XT kit (Illumina Inc. San Diego, CA, USA).	MIC determined by E-test® (Biomerieux) method.	The European Committee on Antimicrobial Susceptibility Testing (EUCAST).	MIC ≤0.5mg/l (susceptible to tetracycline) MIC >1mg/l (resistance to tetracycline).

MIC = minimum inhibitory concentration. NA = not available.

Table 7. Mutations in *pen*A, and *mtr*R genes from studies of resistant isolates.

Author	Year of publication	Mutations *pen*A	Mutations *mtr*R
[14]	2006	D345a	-
[4]	2011	XXXIV; XXXVII; XXXVIII; XXXV	A deletion; G45D; A39T; R44H
[5]	2013	A501T; XVIII	A deletion; H105Y; A39T; G45D; D79N, T86A
[15]	2014	new1; new6; new8; V; XVII; XIII; A501T; A501V; G42S	A deletion; H105Y; A39T; G45D
[16]	2015	XIII	A deletion; A39T;
[17]	2016	XXXIV; XXXVI	A deletion
[7]	2016	XXXIV; A50IV; IV; XIX; XXXVI; P551L; G542S	A deletion; H105Y; G45D; D19N; T86A; A39T
[9]	2017	II; XVII; XII; A375T; A501V; G452S; P551S	A deletion; G45D; A39T; A40D
[18]	2018	IX; II; XII;	A deletion; G45D; H105Y; A39T
[19]	2019	X; VII; XII; XIII; XXVII	A deletion; G45D
[11]	2019	I312M; V316T; N512Y; G545S; A501V; P551S	A deletion; H505Y; G34D; A39T; T86A
[20]	2019	XXXX	A deletion;
[21]	2020	XIV; II; IX; XIX; XXII;	A deletion; G45D; A39T; H205Y; D39N; T86A

Table 8. Mutations in *pen*A, and *mtr*R genes from studies of isolates with reduced susceptibility.

Author	Year of publication	Mutations penA	Mutations mtrR
[1]	2006	F504L; A510V; N512Y; A516G; A509T; P522V; K555Q; I556V; mosaic-1; mosaic-2	-
[2]	2006	Mosaico	A deletion; G45A; Y105H
[3]	2011	A501V; A516G; F404L; P551S; I566V; I556V; K555Q	A deletion; 139T; H105Y; G45D
[4]	2011	XXXIV; XXXVII; XXXVIII; XXXV	A deletion; G45D; A39T; R44H
[5]	2013	A501T; XVIII	A deletion; H105Y; A39T; G45D; D79N, T86A
[6]	2014	XVIII; XLI; XIII;	A deletion
[8]	2017	XVII; XII; XXV; V; XXI; XVII; A501V; G4542S	A deletion; G45D; A39T; A40D
[10]	2017	XXXIV; X; V; XII; XIII	A deletion; H105Y
[9]	2017	II; XVII; XII; A375T; A501V; G452S; P551S	A deletion; G45D; A39T; A40D
[11]	2018	I312M; V316T; N512Y; G545S; A501V; P551S	A deletion; H505Y; G34D; A39T; T86A
[12]	2019	XXXIV; A501T	-
[13]	2019	D345a	A deletion; T insertion

Table 9. Mutations in the *mtr*R gene.

Author	Year of publication	*mtr*R mutations
[2]	2006	A deletion; D95D; Y105H
[3]	2011	A39T; H105Y; G45D; T86A;
[4]	2011	A deletion; G45D; A39T
[5]	2013	A deletion; A39T; H105Y; D79N; T86A; G45D
[15]	2014	A deletion; H105Y; 545D; A39T
[7]	2016	A deletion; H105Y; G24D;
[23]	2016	A deletion; A39T; R44H; A77P; H105Y; D76A; D79N; F178L; A142S
[8]	2017	A deletion; G45D; A39T; A40D
[9]	2017	A deletion; G45D
[10]	2017	A deletion; H105Y
[18]	2018	A deletion; G45D; H105Y; A39T
[11]	2018	A deletion; G45D A39T; H105Y
[25]	2019	A deletion; G45D;
[20]	2019	A deletion; T insertion; G45D; A39T
[24]	2019	A deletion; A39T; G45D
[13]	2019	No mutations
[26]	2020	A deletion; A39T
[21]	2020	A deletion; A39T; G45D; T86A; D79N; H105Y

Table 10. Mutations in the *gyr*A and *par*C genes.

Autor	Year of publication	*gyr*A	*par*C
[27]	1999	S91F	D86N; E91K; R116L; S87N; G85C
[28]	2000	S91F; D95N; D95G	D86N; S87N; E91K
[29]	2000	S91F; D95N; D95G;	S88P; S87R; E91G
[30]	2000	S91F; A67S; A75S; D95G; D95N;	S88P; R116H; A92G; E91G; E91Q; E91K;
[31]	2002	S91F; D95N; D95G	E91G; F100Y;
[32]	2003	S91F; D95G	D86N; S87R; L131L; Y104Y
[33]	2004	S91F; D95N; D95G; A92P; D95Y;	D86N; S88P; E91G; S87N; S87I
[34]	2004	S91F; D95G;	D86N; S87R; E91G
[35]	2004	S91F; D95G;	S87R; E91G
[36]	2004	S91F; D95N; D95G	D86N; S87R; S88P
[37]	2004	S91F; D95N	D86N; E91G
[38]	2004	S91F; D95N; D95A; D95G	D86N; S87R; S87N; S88P; E91A; E91G;
[39]	2006	S91F; A92P; D95A; D95G	S87N; S87R; E91A; E91G
[40]	2006	S91F; D95A; D95G; D95N	D86N; S87N; E91G; S87I
[41]	2008	S91F; D95N; D95A; D95G	D87N
[42]	2010	S91F; D95N; D95A; D95G	S87R; S87N; E91A; D86N
[4]	2011	S91F; D95G; D95A	S87R; S87N; E91G; E91Q
[43]	2011	S91F; D95G; Q102H	D86N; S87R; E91K; E91Q; A92G
[44]	2012	S91F; D95N; D95G	E91G
[45]	2013	S91F; D95N; D96G	D86N; S87N; S87I; S87R; E91G; E91K; E91Q
[20]	2019	S91F; D95A; D95G	S87N; S87R; E91G; D86N;
[46]	2019	S91F; D95G; D95A	E91G
[47]	2019	S91F; D95G	S87R; E91G; S87N; D86N

REFERENCES

[1] Takahata, S., Senju, N., Osaki, Y., Yoshida, T., Ida, T., 2006. Amino acid substitutions in mosaic penicillin-binding protein 2 associated with reduced susceptibility to cefixime in clinical isolates of *Neisseria gonorrhoeae*. *Antimicrob. Agents Chemother.*, 50(11), pp. 3638–3645. doi:10.1128/AAC.00626-06

[2] Tanaka, M., Nakayama, H., Huruya, K., Konomi, I., Irie, S., Kanayama, A., et al., 2006. Analysis of mutations within multiple genes associated with resistance in a clinical isolate of *Neisseria gonorrhoeae* with

reduced ceftriaxone susceptibility that shows a multidrug-resistant phenotype. *Int. J. Antimicrob. Agents*, 27(1), pp. 20–26. doi:10.1016/j.ijantimicag.2005.08.021

[3] Liao, M., Gu, W.M., Yang, Y., Dillon, J.A.R., 2011. Analysis of mutations in multiple loci of *Neisseria gonorrhoeae* isolates reveals effects of PIB, PBP2, and MtrR on reduced susceptibility to ceftriaxone. *J. Antimicrob. Chemother.*, 66(5), pp. 1016–1023. doi:10.1093/jac/dkr021

[4] Allen, V.G., Farrell, D.J., Rebbapragada, A., Tan, J., Tijet, N., Perusini, S.J., et al., 2011. Molecular analysis of antimicrobial resistance mechanisms in *Neisseria gonorrhoeae* isolates from Ontario, Canada. *Antimicrob. Agents Chemother.*, 55(2), pp. 703–712. doi:10.1128/AAC.00788-10

[5] Olsen, B., Lan, P.T., Golparian, D., Johansson, E., Khang, T.H., Unemo, M., 2013. Antimicrobial susceptibility and genetic characteristics of *Neisseria gonorrhoeae* isolates from Vietnam, 2011. *BMC Infect. Dis.*, 13(1). doi:10.1186/1471-2334-13-40

[6] Li, S., Su, X.H., Le, W.J., Jiang, F.X., Wang, B.X., Rice, P.A., 2014. Antimicrobial susceptibility of *Neisseria gonorrhoeae* isolates from symptomatic men attending the Nanjing sexually transmitted diseases clinic (2011-2012): Genetic characteristics of isolates with reduced sensitivity to ceftriaxone. *BMC Infect. Dis.*, 14(1), pp. 1–10. doi:10.1186/s12879-014-0622-0

[7] Carannante, A., Vacca, P., Ghisetti, V., Latino, M.A., Cusini, M., Matteelli, A., et al., 2017. Genetic resistance determinants for cefixime and molecular analysis of gonococci isolated in Italy. *Microb. Drug Resist.*, 23(2), pp. 247–252. doi:10.1089/mdr.2016.0086

[8] Jiang, F.X., Lan, Q., Le, W.J., Su, X.H., 2017. Antimicrobial susceptibility of *Neisseria gonorrhoeae* isolates from Hefei (2014-2015): Genetic characteristics of antimicrobial resistance. *BMC Infect. Dis.*, 17(1), pp. 1–6. doi:10.1186/s12879-017-2472-z

[9] Peng, T., Lin, H., Liu, Q., Cao, W., Ding, H., Chen, J., et al., 2017. Ceftriaxone susceptibility and molecular characteristics of *Neisseria gonorrhoeae* isolates in Changsha, China. *J. Infect. Chemother.*, 23(6), pp. 385–389. doi:10.1016/j.jiac.2017.03.007

[10] Gianecini, R., Romero, M.D.L.M., Oviedo, C., Vacchino, M., Galarza, P., 2017. Emergence and spread of *Neisseria gonorrhoeae* isolates with decreased susceptibility to extended-spectrum cephalosporins in Argentina, 2009 to 2013. *Sex. Transm. Dis.*, 44(6), pp. 351–355. doi:10.1097/OLQ.0000000000000603

[11] Jamaldin, N., Gedye, K., Collins-Emerson, J., Benschop, J., & Nulsen, M. (2019). Phenotypic and genotypic characterization of *Neisseria gonorrhoeae* isolates from New Zealand with reduced susceptibility to ceftriaxone. *Microbial Drug Resistance*, 25(7), 1003–1011. doi:10.1089/mdr.2018.0111

[12]De Laat, M.M., Wind, C.M., Bruisten, S.M., Dierdorp, M., De Vries, H.J.C., Schim Van Der Loeff, M.F., et al. (2019). Ceftriaxone reduced susceptible *Neisseria gonorrhoeae* in the Netherlands, 2009 to 2017: From PenA mosaicism to A501T/V nonmosaicism. *Sexually Transmitted Diseases*, 46(9), 594–601. doi10.1097/OLQ.0000000000001031

[13] Shaskolskiy, B., Dementieva, E., Kandinov, I., Filippova, M., Petrova, N., Plakhova, X., et al. (2019). Resistance of *Neisseria gonorrhoeae* isolates to beta-lactam antibiotics (benzylpenicillin and ceftriaxone) in Russia, 2015-2017. *PLOS One*, 14(7), e0220339. doi10.1371/journal.pone.0220339

[14] Malakhova, M.V., Vereshchagin, V.A., Ilina, E.N., Govorun, V.M., Zubkov, M.M., Priputnevich, T.V., et al. (2006). Analysis of genetic markers of *Neisseria gonorrhoeae* resistance to β-lactam antibiotics. *Bulletin of Experimental Biology and Medicine*, 141(5), 610–615. doi10.1007/s10517-006-0234-9

[15] Chen, S., et al. (2014). Antimicrobial resistance, genetic resistance determinants for ceftriaxone, and molecular epidemiology of *Neisseria gonorrhoeae* isolates in Nanjing, China. *Journal of Antimicrobial Chemotherapy*, 69(12), 2959–2965. doi:10.1093/jac/dku245

[16] Lee, H., Unemo, M., Kim, H.J., Seo, Y., Lee, K., Chong, Y. (2015). Emergence of decreased susceptibility and resistance to extended-spectrum cephalosporins in *Neisseria gonorrhoeae* in Korea. *Journal of Antimicrobial Chemotherapy*, 70(9), 2536–2542. doi:10.1093/jac/dkv146

[17] De Curraize, C., Sylvain, K., Micaëlo, M., Fournet, N., Ruche, G.L., Meunier, F., Amarsy-Guerle, R., Jacquier, H., Cambau, E., Bercot, B. (2016). Ceftriaxone-resistant *Neisseria gonorrhoeae* isolates (2010 to 2014) in France characterized by using whole-genome sequencing. *Antimicrobial Agents and Chemotherapy*, 60(11), 9–13. doi:10.1128/AAC.01568-16

[18] Thakur, S.D., Levett, P.N., Horsman, G.B., Dillon, J.A.R. (2018). Association of *Neisseria gonorrhoeae* genogroups and specific PBP2/MtrR/PorB mutation patterns with susceptibility to penicillin in a susceptible gonococcal population. *Journal of Antimicrobial Chemotherapy*, 73(10), 2682–2686. doi:10.1093/jac/dky233

[19] Yan, J., et al. (2019). Increasing prevalence of *Neisseria gonorrhoeae* with decreased susceptibility to ceftriaxone and resistance to azithromycin in Hangzhou, China (2015-17). *Journal of Antimicrobial Chemotherapy*, 74(1), 29–37. doi:10.1093/jac/dky412

[20] Calado, J., Castro, R., Lopes, Â., Campos, M.J., Rocha, M., Pereira, F. (2019). Antimicrobial resistance and molecular characteristics of *Neisseria gonorrhoeae* isolates from men who have sex with men. *International Journal of Infectious Diseases*, 79, 116–122. doi:10.1016/j.ijid.2018.10.030

[21] Kivata, M.W., Mbuchi, M., Eyase, F., Bulimo, W.D., Kyanya, C.K., Oundo, V., et al., 2020. Plasmid mediated penicillin and tetracycline resistance among *Neisseria gonorrhoeae* isolates from Kenya. *BMC Infectious Diseases*, 20(1), pp.1–11. doi:10.1186/s12879-020-05398-5.

[22] Chen, P.L., Lee, H.C., Yan, J.J., Hsieh, Y.H., Lee, N.Y., Ko, N.Y., et al., 2010. High prevalence of mutations in quinolone-resistance-determining regions and *mtrR* loci in polyclonal *Neisseria gonorrhoeae* isolates at a tertiary hospital in Southern Taiwan. *Journal of the Formosan Medical Association*, 109(2), pp.120–127. doi:10.1016/S0929-6646(10)60032-0.

[23] Belkacem, A., Jacquier, H., Goubard, A., Mougari, F., La Ruche, G., Patey, O., et al., 2016. Molecular epidemiology and mechanisms of resistance of azithromycin-resistant *Neisseria gonorrhoeae* isolated in France during 2013-14. *Journal of Antimicrobial Chemotherapy*, 71(9), pp.2471–2478. doi:10.1093/jac/dkw182.

[24] Liu, Y., Wang, Y., Liao, C., Hsueh, P., 2019. Emergence and spread of *Neisseria gonorrhoeae* strains with high-level resistance to azithromycin in Taiwan from 2001 to 2018. *Antimicrobial Agents and Chemotherapy*, 63(9), pp.1–8. doi:10.1128/AAC.00773-19.

[25] Zheng, Z., Liu, L., Shen, X., Yu, J., Chen, L., Zhan, L., et al., 2019. Antimicrobial resistance and molecular characteristics among *Neisseria gonorrhoeae* clinical isolates in a Chinese tertiary hospital. *Infectious Diseases and Therapy*, 12, pp.3301–3309. Doi:10.2147/IDR.S221109.

[26] Maduna, L.D., Kock, M.M., Van der Veer, B.M.J.W., Radebe, O., McIntyre, J., Van Alphen, L.B., et al., 2020. Antimicrobial resistance of *Neisseria gonorrhoeae* isolates from high-risk men in Johannesburg, South Africa. *Antimicrobial Agents and Chemotherapy*, 64(11), pp.1–34. doi:10.1128/AAC.00906-20.

[27] Trees, D.L., Sandul, A.L., Peto-Mesola, V., Aplasca, M.R., Bun Leng, H., Whittington, W.L., et al., 1999. Alterations within the quinolone resistance-determining regions of GyrA and ParC of *Neisseria gonorrhoeae*

isolated in the Far East and the United States. *International Journal of Antimicrobial Agents*, 12(4), pp.325–332. doi:10.1016/S0924-8579(99)00081-3.

[28] De Neeling, A.J., Van Santen-Verheuvel, M., Spaargaren, J., Willems, R.J.L., 2000. Antimicrobial resistance of *Neisseria gonorrhoeae* and emerging ciprofloxacin resistance in The Netherlands, 1991 to 1998. *Antimicrobial Agents and Chemotherapy*, 44(11), pp.3184–3185. doi:10.1128/AAC.44.11.3184-3185.2000.

[29] Tanaka, M., Nakayama, H., Haraoka, M., Saika, T., Kobayashi, I., Naito, S., 2000. Susceptibilities of *Neisseria gonorrhoeae* isolates containing amino acid substitutions in GyrA, with or without substitutions in ParC, to newer fluoroquinolones and other antibiotics. *Antimicrobial Agents and Chemotherapy*, 44(1), pp.192–195. doi:10.1128/AAC.44.1.192-195.2000.

[30] Tanaka, M., Nakayama, H., Haraoka, M., Saika, T., Kobayashi, I., Naito, S., 2000. Antimicrobial resistance of *Neisseria gonorrhoeae* and high prevalence of ciprofloxacin-resistant isolates in Japan, 1993 to 1998. *Journal of Clinical Microbiology*, 38(2), pp.521–525. doi:10.1128/jcm.38.2.521-525.2000.

[31] Chaudhry, K., Ray, M. & Bala, D.S., 2002. Mutation patterns in gyrA and parC genes of ciprofloxacin resistant isolates of *Neisseria gonorrhoeae* from India. *Sexually Transmitted Infections*, 78(6), pp.440–444. doi:10.1136/sti.78.6.440.

[32] Alcalá, L. et al., 2003. Molecular characterization of ciprofloxacin resistance of gonococcal strains in Spain. *Sexually Transmitted Diseases*, 30(5), pp.395–398. doi:10.1097/00007435-200305000-00004.

[33] Saika, T., Kobayashi, I. & Inoue, M., 2004. A comparison of the microbiological characteristics of *Neisseria gonorrhoeae* isolated from male and female patients with gonorrhea. *Chemotherapy*, 50(2), pp.92–97. doi:10.1159/000077809.

[34] Yoo, J. et al., 2004. Antimicrobial resistance patterns (1999–2002) and characterization of ciprofloxacin-resistant *Neisseria gonorrhoeae* in Korea. *Sexually Transmitted Diseases*, 31(5), pp.305–310. doi:10.1097/01.OLQ.0000123650.98303.EB.

[35] Vereshchagin, V.A. et al., 2004. Fluoroquinolone-resistant *Neisseria gonorrhoeae* isolates from Russia: Molecular mechanisms implicated. *Journal of Antimicrobial Chemotherapy*, 53(4), pp.653–656. doi:10.1093/jac/dkh145.

[36] Dewi, B.E., Akira, S., Hayashi, H. & Ba-thein, W., 2004. High occurrence of simultaneous mutations in target enzymes and MtrRCDE efflux system in quinolone-resistant *Neisseria gonorrhoeae*. *Sexually Transmitted Diseases*, 31(6), pp.353–359. doi:10.1097/00007435-200406000-00007.

[37] Uthman, A. et al., 2004. High-frequency of quinolone-resistant *Neisseria gonorrhoeae* in Austria with a common pattern of triple mutations in GyrA and ParC genes. *Sexually Transmitted Diseases*, 31(10), pp.616–618. doi:10.1097/01.olq.0000140019.18390.28.

[38] Giles, J.A. et al., 2004. Quinolone resistance-determining region mutations and por type of *Neisseria gonorrhoeae* isolates: Resistance surveillance and typing by molecular methodologies. *Journal of Infectious Diseases*, 189(11), pp.2085–2093. doi:10.1086/386312.

[39] Yang, Y. et al., 2006. Antimicrobial susceptibility and molecular determinants of quinolone resistance in *Neisseria gonorrhoeae* isolates from Shanghai. *Journal of Antimicrobial Chemotherapy*, 58(4), pp.868–872. doi:10.1093/jac/dkl301.

[40] Wang, B. et al., 2006. Antimicrobial susceptibility of *Neisseria gonorrhoeae* isolated in Jiangsu Province, China, with a focus on fluoroquinolone resistance. *Journal of Medical Microbiology*, 55(9), pp.1251–1255. doi:10.1099/jmm.0.46401-0.

[41] Ilina, E.N., Vereshchagin, V.A., Borovskaya, A.D., Malakhova, M.V., Sidorenko, S.V., Al-Khafaji, N.C., et al. (2008) 'Relation between genetic markers of drug resistance and susceptibility profile of clinical *Neisseria gonorrhoeae* strains', *Antimicrobial Agents and Chemotherapy*, 52(6), pp. 2175–2182. doi:10.1128/AAC.01420-07.

[42] Chen, P.L., Lee, H.C., Yan, J.J., Hsieh, Y.H., Lee, N.Y., Ko, N.Y., et al. (2010) 'High Prevalence of Mutations in Quinolone-resistance-determining Regions and mtrR Loci in Polyclonal *Neisseria gonorrhoeae* Isolates at a Tertiary Hospital in Southern Taiwan', *Journal of Formosan Medical Association*, 109(2), pp. 120–127. doi:10.1016/S0929-6646(10)60032-0.

[43] Uehara, A.A., Amorin, E.L.T., Ferreira, M.D.F., Andrade, C.F., Clementino, M.B.M., De Filippis, I., et al. (2011) 'Molecular characterization of quinolone-resistant *Neisseria gonorrhoeae* isolates from Brazil', *Journal of Clinical Microbiology*, 49(12), pp. 4208–4212. doi:10.1128/JCM.01175-11.

[44] Kulkarni, S., Bala, M., Sane, S., Pandey, S., Bhattacharya, J., Risbud, A. (2012) 'Mutations in the gyrA and parC genes of quinolone-resistant *Neisseria gonorrhoeae* isolates in India', *International Journal of Antimicrobial Agents*, 40(6), pp. 549–553. doi:10.1016/j.ijantimicag.2012.08.007.

[45] Sethi, S., Golparian, D., Bala, M., Dorji, D., Ibrahim, M., Jabeen, K., et al. (2013) 'Antimicrobial susceptibility and genetic characteristics of *Neisseria gonorrhoeae* isolates from India, Pakistan and Bhutan in 2007-2011', *BMC Infectious Diseases*, 13(1), pp. 1–8. doi:10.1016/j.ijantimicag.2012.08.007.

[46] Kivata, M.W., Mbuchi, M., Eyase, F.L., Bulimo, W.D., Kyanya, C.K., Oundo, V., et al. (2019) 'GyrA and parC mutations in fluoroquinolone-resistant *Neisseria gonorrhoeae* isolates from Kenya', *BMC Microbiology*, 19(1), pp. 1–9. doi:10.1186/s12866-019-1439-1.

[47] Boiko, I., Golparian, D., Jacobsson, S., Krynytska, I., Frankenberg, A., Shevchenko, T., et al. (2020) 'Genomic epidemiology and antimicrobial resistance determinants of *Neisseria gonorrhoeae* isolates from Ukraine, 2013–2018', *Apmis*, 128(7), pp. 465–475. doi:10.1111/apm.13060.

[48] Chalkley, L.J., Janse Van Rensburg, M.N., Matthee, P.C., Ison, C.A., Botha, P.L. (1997) 'Plasmid analysis of *Neisseria gonorrhoeae* isolates and dissemination of tetM genes in southern Africa 1993-1995', *Journal of Antimicrobial Chemotherapy*, 40(6), pp. 817–822. doi:10.1093/jac/40.6.817.

[49] Dyck, E., Alary, M., Guédou, A., Abdellati, S., Lafia, E., Anagonou, S. (2001) 'Antimicrobial susceptibilities and plasmid patterns of *Neisseria gonorrhoeae* in Bénin', *International Journal of STD & AIDS*, 12(2), pp. 89–93. doi:10.1258/0956462011916848.

[50] Dillon, J.A.R., Li, H., Sealy, J., Ruben, M., Prabhakar, P., Swantson, W.H., et al. (2001) 'Antimicrobial susceptibility of *Neisseria gonorrhoeae* isolates from three Caribbean countries: Trinidad, Guyana, and St. Vincent', *Sexually Transmitted Diseases*, 28(9), pp. 508–514. doi:10.1097/00007435-200109000-00006.

[51] Dillon, J.A., Rubabaza, J.P., Benzaken, A.S., Sardinha, J.C., Li, H., Bandeira, M.G., Fernando Filho, E. dos Santos, 2001. Reduced susceptibility to azithromycin and high percentages of penicillin and tetracycline resistance in *Neisseria gonorrhoeae* isolates from Manaus, Brazil, 1998. *Sexually Transmitted Diseases*, 28(9), pp.521-526. doi:10.1097/00007435-200109000-00008

[52] Márquez, C.M., Dillon, J.A.R., Rodriguez, V., Borthagaray, G., 2002. Detection of a novel *tetM* determinant in tetracycline-resistant *Neisseria gonorrhoeae* from Uruguay, 1996-1999. *Sexually Transmitted Diseases*, 29(12), pp.792-797. doi:10.1097/00007435-200212000-00010

[53] Su, X., Jiang, F., Qimuge, X., Dai, X., Sun, H., Ye, S., 2007. Surveillance of antimicrobial susceptibilities in *Neisseria gonorrhoeae* in Nanjing, China, 1999-2006. *Sexually Transmitted Diseases*, 34(12), pp.995-999.doi:10.1097/OLQ.0b013e3180ca8f24

[54] Karim, S., Bouchikhi, C., Banani, A., El Fatemi, H., Souho, T., Erraghay, S., et al., 2018. Molecular antimicrobial resistance of *Neisseria gonorrhoeae* in a Moroccan area. *Infectious Diseases in Obstetrics and Gynecology*, 2018. doi:10.1155/2018/7263849

[55] Rambaran, S., Naidoo, K., Dookie, N., Moodley, P., Sturm, A.W., 2019. Resistance profile of *Neisseria gonorrhoeae* in KwaZulu-Natal, South Africa questioning the effect of the currently advocated dual therapy. *Sexually Transmitted Diseases*, 46(4), pp.266-270. doi:10.1093/jac/40.6.817

[56] Kivata, M.W., Mbuchi, M., Eyase, F., Bulimo, W.D., Kyanya, C.K., Oundo, V., et al., 2020. Plasmid mediated penicillin and tetracycline resistance among *Neisseria gonorrhoeae* isolates from Kenya. *BMC Infectious Diseases*, 20(1), pp.1-11. doi:10.1186/s12879-020-05398-5

N. gonorrhoeae and antibiotic resistance
