# Supplementary material for: The frequency of mutations in the penA, mtrR, gyrA and parC genes of Neisseria gonorrhoeae, the presence of tetM gene and antibiotic resistance/susceptibility: a systematic review and meta-analyses
Source: Front Microbiol. 2025 Jan 22;15:1414330. doi: 10.3389/fmicb.2024.1414330 (PMC11808683; doi:10.3389/fmicb.2024.1414330)
Supplement: Supplementary file 2 [file Image_1.pdf]

SUPPLEMENTARY MATERIAL - FIGURES

The frequency of mutations in the *pen*A, *mtr*R, *gyr*A and *par*C genes of *Neisseria gonorrhoeae*, the presence of *tet*M gene and antibiotic resistance/susceptibility: a systematic review and meta-analyses

[+]Ana Clara Mendes, Renan Pedra de Souza[#], Diana Bahia[#]

Departamento de Genética, Ecologia e Evolução, Instituto de Ciências Biológicas, Universidade Federal de Minas Gerais, Brazil.

SUPPLEMENTARY MATERIAL - FIGURES

FIGURES-SUPPLEMENTARY MATERIAL

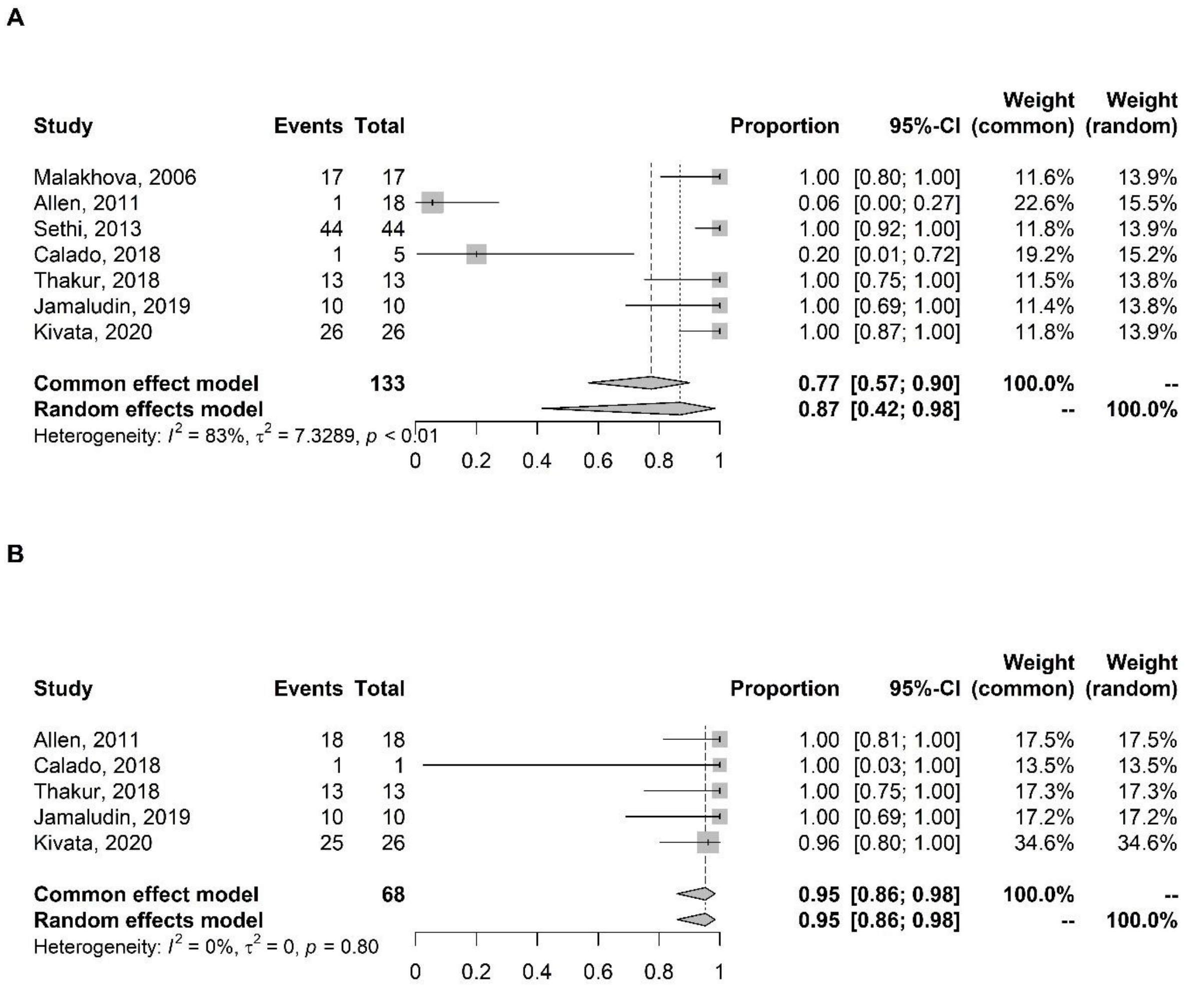

Figure 1. Meta-analysis of the proportion of gene mutations in isolates resistant to the antibiotic penicillin. (A) *pen*A. (B) *mtr*R. The results are presented in forest plots. In its first column ("study") are the citations of the selected studies. The second column ("events") contains the number of isolates with mutations in the evaluated gene, while the third column ("total") shows the total number of samples analyzed in that study. The fourth column presents the proportion estimate followed by their confidence intervals. The sixth and seventh columns present, respectively, the weights for the fixed and random models of the meta-analysis.

SUPPLEMENTARY MATERIAL - FIGURES

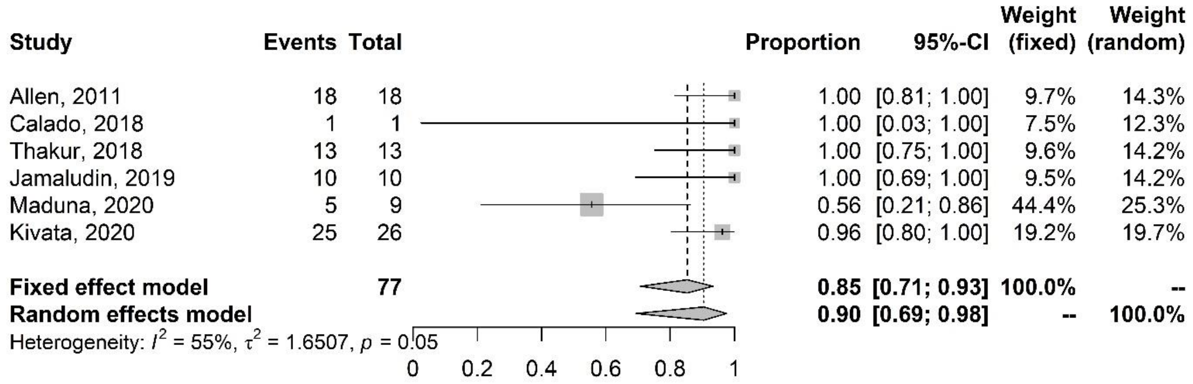

Figure. 2. Meta-analysis of the proportion of gene mutations in isolates resistant to penicillin and *mtr*R gene. The results are presented in forest plots. In its first column ("study") are the citations of the selected studies. The second column ("events") contains the number of isolates with mutations in the evaluated gene, while the third column ("total") shows the total number of samples analyzed in that study. The fourth column presents the proportion estimate followed by their confidence intervals. The sixth and seventh columns present, respectively, the weights for the fixed and random models of the meta-analysis.

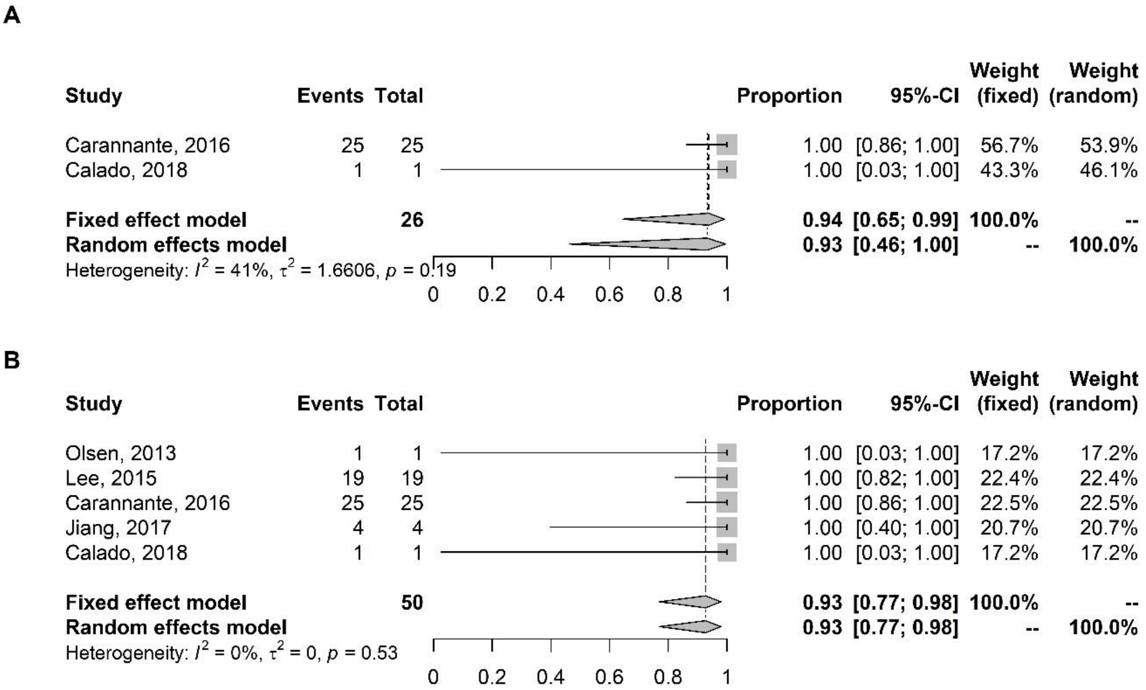

Figure. 3. Meta-analysis of the proportion of gene mutations in cefixime-resistant isolates. (A) *mtr*R. (B) *pen*A. The results are presented in forest plots. In its first column ("study") are the citations of the selected studies. The second column ("events") contains the number of isolates with mutations in the evaluated gene, while the third column ("total") shows the total number of samples analyzed in that study. The fourth column presents the proportion estimate followed by their confidence intervals. The sixth and seventh columns present, respectively, the weights for the fixed and random models of the meta-analysis.

SUPPLEMENTARY MATERIAL - FIGURES

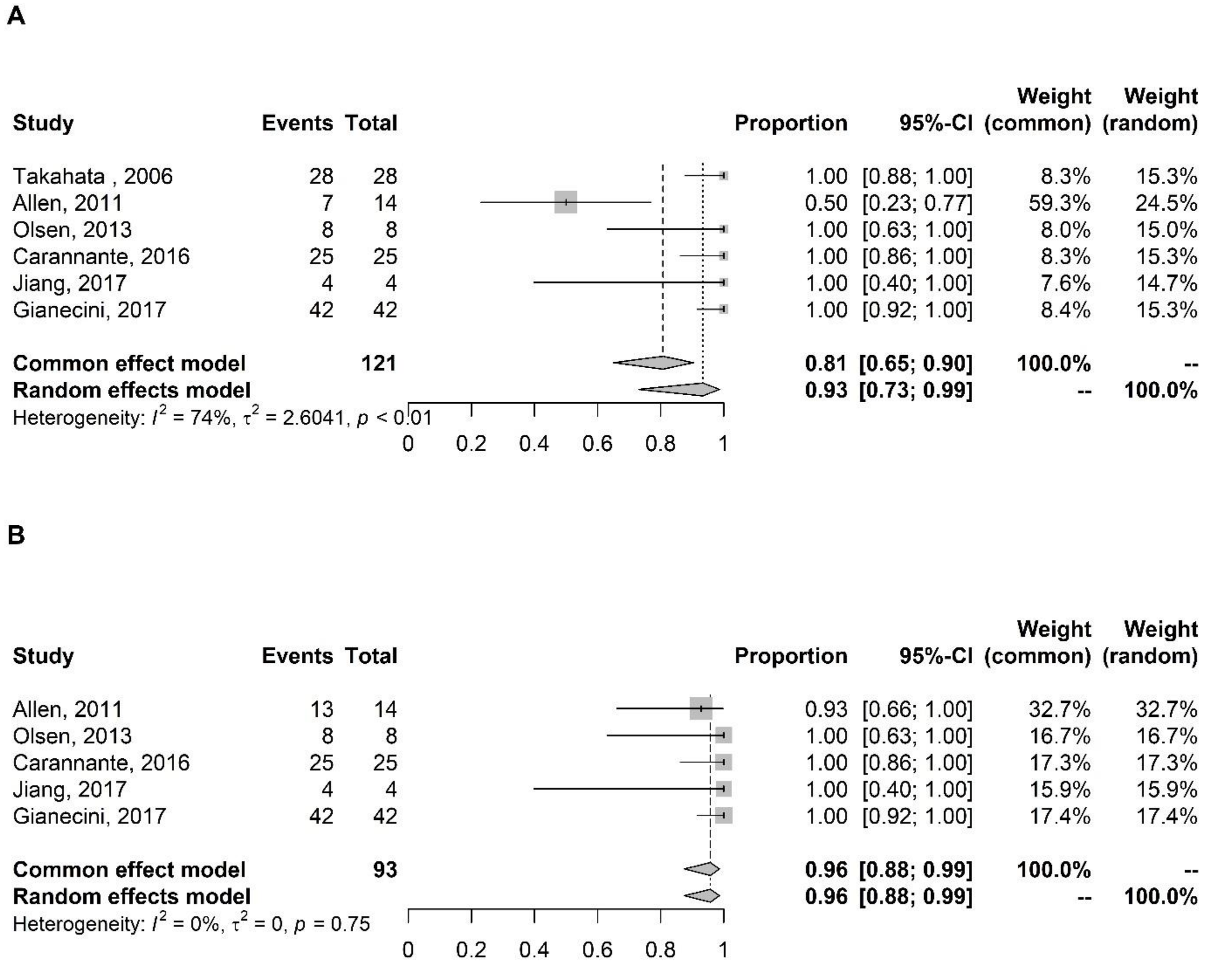

Figure 4. Meta-analysis of the proportion of gene mutations in isolates with reduced susceptibility to cefixime. (A) *pen*A. (B) *mtr*R. The results are presented in forest plots. In its first column ("study") are the citations of the selected studies. The second column ("events") contains the number of isolates with mutations in the evaluated gene, while the third column ("total") shows the total number of samples analyzed in that study. The fourth column presents the proportion estimate followed by their confidence intervals. The sixth and seventh columns present, respectively, the weights for the fixed and random models of the meta-analysis.

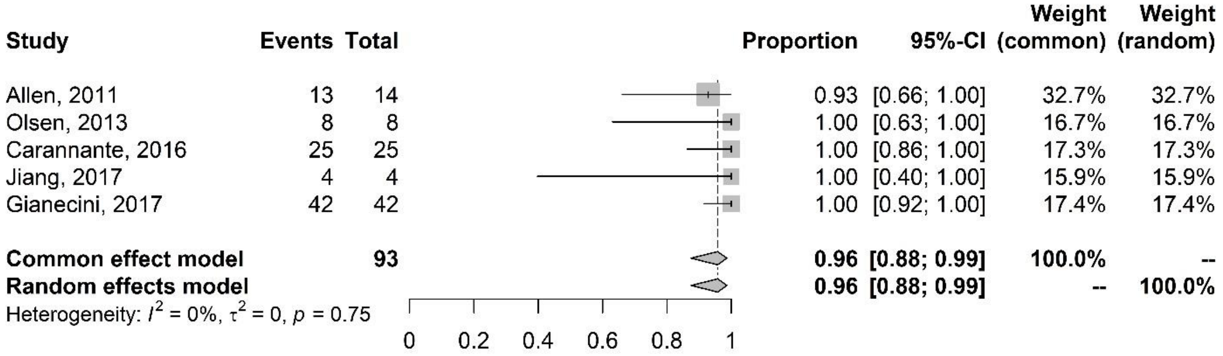

Figure. 5. Meta-analysis of the proportion of gene mutations in isolates with reduced susceptibility to cefixime (*mtr*R gene). The results are presented in forest plots. In its first column ("study") are the citations of the selected studies. The second column ("events") contains the number of isolates with mutations in the evaluated gene, while the third column ("total") shows the total number of samples analyzed in that study. The fourth column presents the proportion estimate followed by their confidence intervals. The sixth and seventh columns present, respectively, the weights for the fixed and random models of the meta-analysis.

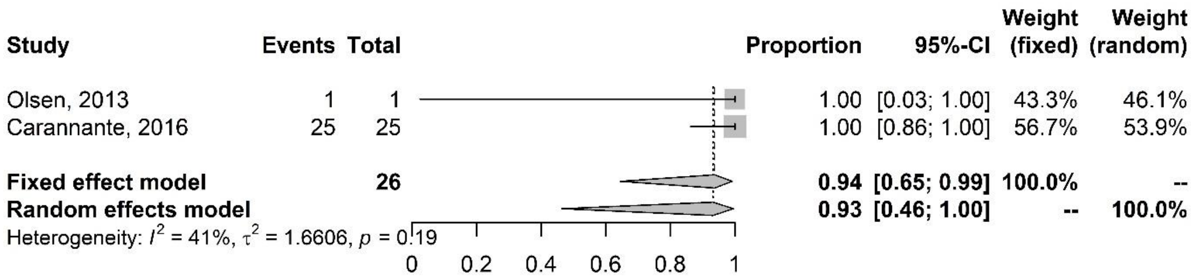

Figure. 6. Meta-analysis of the proportion of gene mutations in cefixime resistant isolates (*mtr*R gene). The results are presented in forest plots. In its first column ("study") are the citations of the selected studies. The second column ("events") contains the number of isolates with mutations in the evaluated gene, while the third column ("total") shows the total number of samples analyzed in that study. The fourth column presents the proportion estimate followed by their confidence intervals. The sixth and seventh columns present, respectively, the weights for the fixed and random models of the meta-analysis.

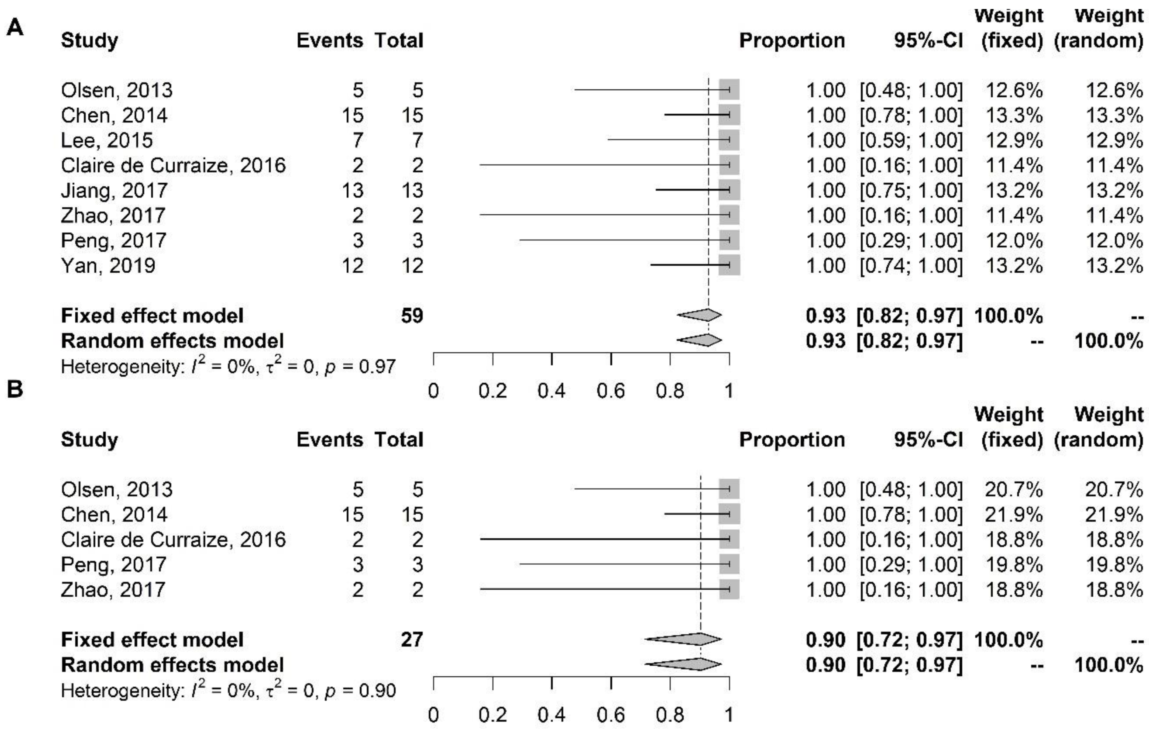

Figure. 7. Meta-analysis of the proportion of gene mutations in ceftriaxone-resistant isolates. (A) *pen*A. (B) *mtr*R. The results are presented in forest plots. In its first column ("study") are the citations of the selected studies. The second column ("events") contains the number of isolates with mutations in the evaluated gene, while the third column ("total") shows the total number of samples analyzed in that study. The fourth column presents the proportion estimate followed by their confidence intervals. The sixth and seventh columns present, respectively, the weights for the fixed and random models of the meta-analysis.

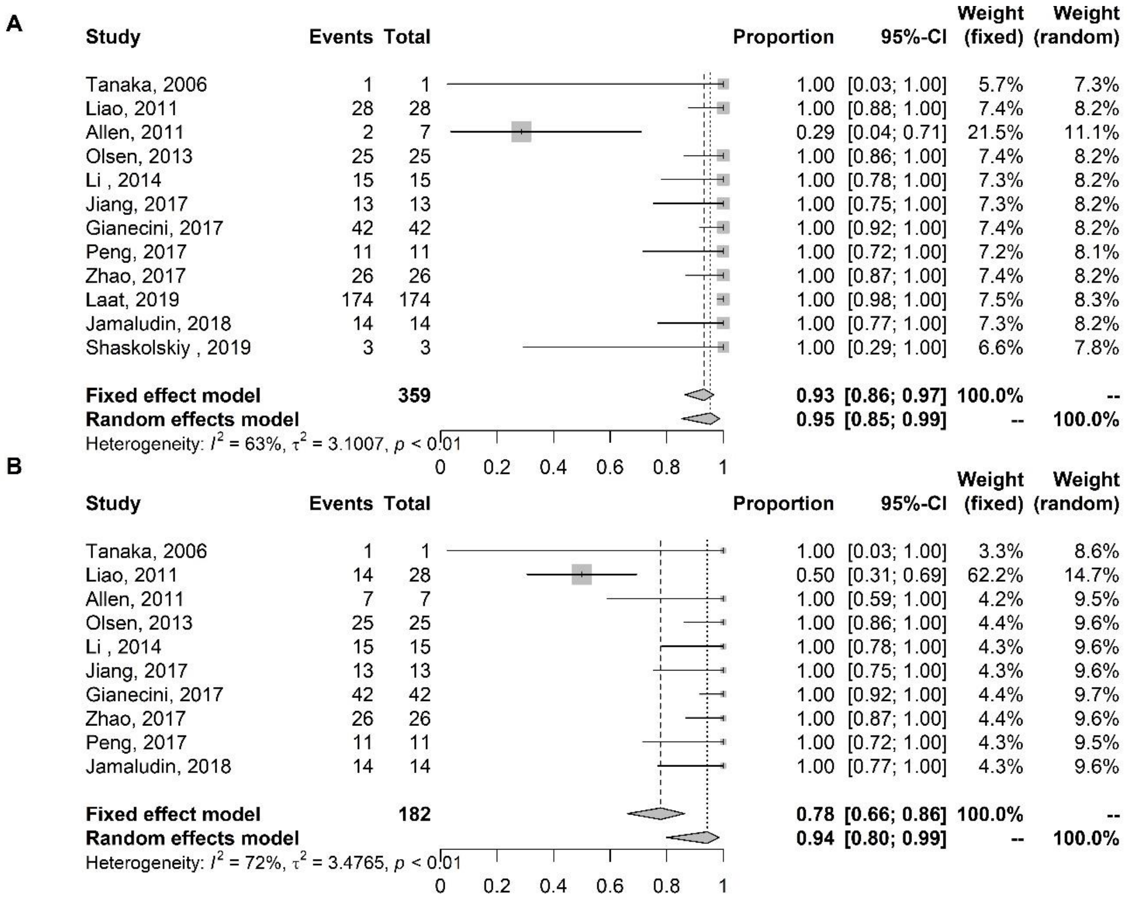

Figure. 8. Meta-analysis of the proportion of gene mutations in isolates with reduced susceptibility to ceftriaxone. (A) *pen*A. (B) *mtr*R. The results are presented in forest plots. In its first column ("study") are the citations of the selected studies. The second column ("events") contains the number of isolates with mutations in the evaluated gene, while the third column ("total") shows the total number of samples analyzed in that study. The fourth column presents the proportion estimate followed by their confidence intervals. The sixth and seventh columns present, respectively, the weights for the fixed and random models of the meta-analysis.

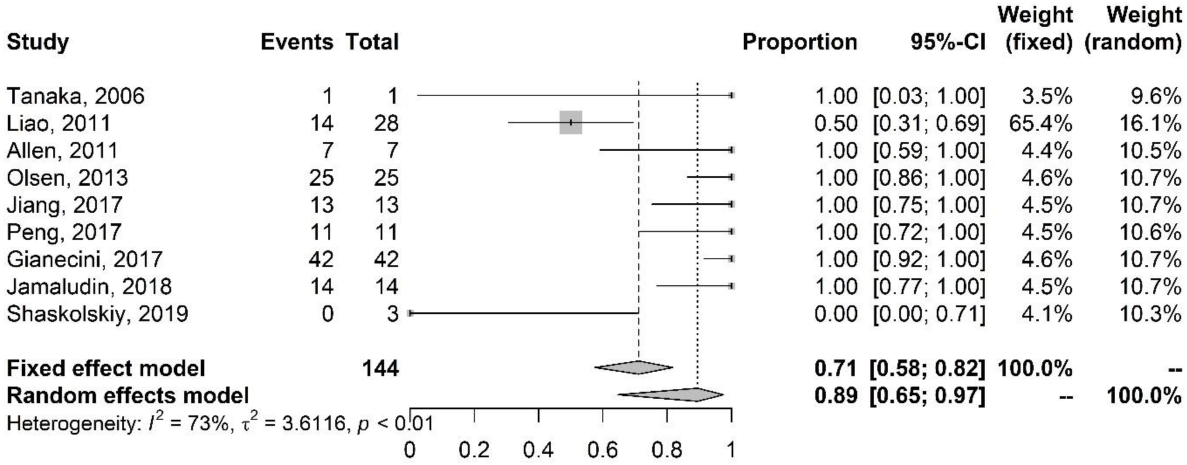

Figure. 9. Meta-analysis of the proportion of gene mutations in isolates with reduced susceptibility to ceftriaxone (*mtr*R gene). The results are presented in forest plots. In its first column ("study") are the citations of the selected studies. The second column ("events") contains the number of isolates with mutations in the evaluated gene, while the third column ("total") shows the total number of samples analyzed in that study. The fourth column presents the proportion estimate followed by their confidence intervals. The sixth and seventh columns present, respectively, the weights for the fixed and random models of the meta-analysis.

SUPPLEMENTARY MATERIAL - FIGURES

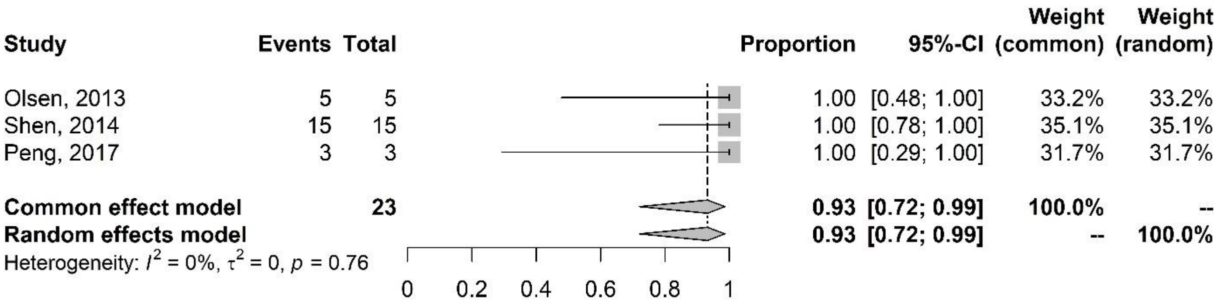

Figure. 10. Meta-analysis of the proportion of gene mutations in isolates resistant to ceftriaxone (*mtr*R gene). The results are presented in forest plots. In its first column ("study") are the citations of the selected studies. The second column ("events") contains the number of isolates with mutations in the evaluated gene, while the third column ("total") shows the total number of samples analyzed in that study. The fourth column presents the proportion estimate followed by their confidence intervals. The sixth and seventh columns present, respectively, the weights for the fixed and random models of the meta-analysis.

SUPPLEMENTARY REFERENCES

[1] M.V. Malakhova, V.A. Vereshchagin, E.N. Ilina, V.M. Govorun, M.M. Zubkov, Priputnevich T V., et al. Analysis of genetic markers of *N. Gonorrhoeae* resistance to β-lactam antibiotics. Bull Exp Biol Med. (2006) 141(5):610–5. https://doi.org/doi/110.1007/s10517-006-0234-9.

[2] V.G. Allen, D.J. Farrell, A. Rebbapragada, J. Tan, N. Tijet, S.J. Perusini, et al. Molecular analysis of antimicrobial resistance mechanisms in *Neisseria gonorrhoeae* isolates from Ontario, Canada. Antimicrob Agents Chemother. (2011) 55(2):703–12. https://doi.org/doi/10.1128/AAC.00788-10.

[3] S. Sethi, D. Golparian, M. Bala, D. Dorji, M. Ibrahim, K. Jabeen., et al. Antimicrobial susceptibility and genetic characteristics of *Neisseria gonorrhoeae* isolates from India, Pakistan and Bhutan in 2007-2011. BMC Infect Dis (2013) 13(1):1. https://doi.org/doi/10.1016/j.ijantimicag.2012.08.007.

[4] J. Calado, R. Castro, Â. Lopes, M.J. Campos, M. Rocha, F. Pereira. Antimicrobial resistance and molecular characteristics of *Neisseria gonorrhoeae* isolates from men who have sex with men. Int J Infect Dis (2019) 79:116–22. https://doi.org/doi/10.1016/j.ijid.2018.10.030.

[5] S.D. Thakur, P.N. Levett, G.B. Horsman, J.A.R. Dillon. Association of *Neisseria gonorrhoeae* genogroups and specific PBP2/MtrR/PorB mutation patterns with susceptibility to penicillin in a susceptible gonococcal population. J Antimicrob Chemother. (2018) 73(10):2682–6. https://doi.org/doi/110.1093/jac/dky233.

[6] N. Jamaludin, K. Gedye, J. Collins-Emerson, J. Benschop, M. Nulsen. Phenotypic and Genotypic Characterization of *Neisseria gonorrhoeae* Isolates from New Zealand with Reduced Susceptibility to Ceftriaxone. Microb Drug Resist. (2019) 25(7):1003–11. https://doi.org/doi/110.1089/mdr.2018.0111.

[7] M.W. Kivata, M. Mbuchi, F. Eyase, W.D. Bulimo, C.K. Kyanya, V. Oundo., et al. Plasmid mediated penicillin and tetracycline resistance among *Neisseria gonorrhoeae* isolates from Kenya. BMC Infect Dis. (2020) 20(1):1–11. https://doi.org/doi/10.1186/s12879-020-05398-5.

[8] L.D. Maduna, M.M. Kock, B.M.J.W. van der Veer, O. Radebe, J. McIntyre, L.B. van Alphen LB, et al. Antimicrobial resistance of *Neisseria gonorrhoeae* isolates from high-risk men in johannesburg, South Africa. Antimicrob Agents Chemother. (2020) 64(11) 1–34. https://doi.org/doi/10.1128/AAC.00906-20.

SUPPLEMENTARY MATERIAL - FIGURES

[9] A. Carannante, P. Vacca, V. Ghisetti, M.A. Latino, M. Cusini, A. Matteelli, et al. Genetic Resistance Determinants for Cefixime and Molecular Analysis of Gonococci Isolated in Italy. Microb Drug Resist. (2017) 23(2):247–52. https://doi.org/doi/10.1089/mdr.2016.0086.

[10] B. Olsen, P.T. Lan, D. Golparian, E. Johansson, T.H. Khang, M. Unemo M. Antimicrobial susceptibility and genetic characteristics of *Neisseria gonorrhoeae* isolates from Vietnam, 2011. BMC Infect Dis. (2013) 13(1). https://doi.org/doi/10.1186/1471-2334-13-40.

[11] H. Lee, M. Unemo, H.J. Kim, Y. Seo, K. Lee, Y. Chong Y. Emergence of decreased susceptibility and resistance to extendedspectrum cephalosporins in *Neisseria gonorrhoeae* in Korea. J Antimicrob Chemother. (2015) 70(9):2536–42. https://doi.org/doi/10.1093/jac/dkv146.

[12] F.X. Jiang, Q. Lan, W.J. Le, X.H. Su. Antimicrobial susceptibility of Neisseria gonorrhoeae isolates from Hefei (2014-2015): Genetic characteristics of antimicrobial resistance. BMC Infect Dis. (2017) 17(1):1–6. https://doi.org/doi/10.1186/s12879-017-2472-z.

[13] S. Takahata, N. Senju, Y. Osaki, T. Yoshida, T. Ida T. Amino acid substitutions in mosaic penicillin-binding protein 2 associated with reduced susceptibility to cefixime in clinical isolates of *Neisseria gonorrhoea*. Antimicrob Agents Chemother. (2006) 50(11):3638–45. https://doi.org/doi/10.1128/AAC.00626-06.

[14] R. Gianecini, M. De Las Mercedes Romero, C. Oviedo, M. Vacchino, P. Galarza. Emergence and spread of *Neisseria gonorrhoeae* isolates with decreased susceptibility to extended-spectrum cephalosporins in Argentina, 2009 to 2013. Sex Transm Dis. (2017) 44(6):351–5. https://doi.org/doi/10.1097/OLQ.0000000000000603.

[15] S. CHEN, et al. Antimicrobial resistance, genetic resistance determinants for ceftriaxone and molecular epidemiology of *Neisseria gonorrhoeae* isolates in Nanjing, China. **Journal of Antimicrobial Chemotherapy**, v. 69, (2014) 2959–2965. https://doi.org/10.1093/jac/dku245.

[16] C. de Curraize, K. Sylvain, M. Micaëlo, N. Fournet, G.L. Ruche, F. Meunier, R. Amarsy-Guerle, H. Jacquier, E. Cambau, B. Bercotb. Laboratory. Ceftriaxone-Resistant *Neisseria gonorrhoeae* Isolates (2010 to 2014) in France Characterized by Using Whole-Genome Sequencing. (2016) 60(11):9–13. https://doi.org/10.1128/AAC.01568-16.

[17] L. Zhao, A. Liu, R. Li, S. Zhao. Trends in antimicrobial resistance in *Neisseria gonorrhoeae and* molecular characteristics of N. gonorrhoeae with decreased susceptibility to ceftriaxone in Shandong, China, 2007 to 2014. Int J Antimicrob Agents. (2018) 51(1):52-56. https://doi.org/10.1016/j.ijantimicag.2017.06.004.

[18] T. Peng, H. Lin, Q. Liu, W. Cao, H. Ding, J. Chen, et al. Ceftriaxone susceptibility and molecular characteristics of *Neisseria gonorrhoeae* isolates in Changsha, China. J Infect Chemother (2017) 23(6):385–9. https://doi.org/10.1016/j.jiac.2017.03.007.

[19] J. Yan. et al. Increasing prevalence of *Neisseria gonorrhoeae* with decreased susceptibility to ceftriaxone and resistance to azithromycin in Hangzhou, China (2015-17). Journal of Antimicrobial Chemotherapy, 74 (2019) 29–37. https://doi.org/10.1093/jac/dky412.

[20] M. Tanaka, H. Nakayama, K. Huruya, I. Konomi, S. Irie, A. Kanayama, et al. Analysis of mutations within multiple genes associated with resistance in a clinical isolate of *Neisseria gonorrhoeae* with reduced ceftriaxone susceptibility that shows a multidrug-resistant phenotype. Int J Antimicrob Agents. (2006) 27(1):20–6. https://doi.org/10.1016/j.ijantimicag.2005.08.021.

[21] M. Liao, W.M. Gu, Y. Yang, J.A.R. Dillon. Analysis of mutations in multiple loci of *Neisseria gonorrhoeae* isolates reveals effects of PIB, PBP2 and MtrR on reduced susceptibility to ceftriaxone. J Antimicrob Chemother. (2011) 66(5):1016–23. https://doi.org/10.1093/jac/dkr021.

SUPPLEMENTARY MATERIAL - FIGURES

[22] S. Li, X.H. Su, W.J. Le, F.X. Jiang, B.X. Wang, P.A. Rice. Antimicrobial susceptibility of *Neisseria gonorrhoeae* isolates from symptomatic men attending the Nanjing sexually transmitted diseases clinic (2011-2012): Genetic characteristics of isolates with reduced sensitivity to ceftriaxone. BMC Infect Dis. (2014) 14(1):1–10. https://doi.org/10.1186/s12879-014-0622-0.

[23] M.M. De Laat, C.M. Wind, S.M. Bruisten, M. Dierdorp, H.J.C. De Vries, M.F. Schim Van Der Loeff, et al. Ceftriaxone Reduced Susceptible *Neisseria gonorrhoeae* in the Netherlands, 2009 to 2017: From PenA Mosaicism to A501T/V Nonmosaicism. Sex Transm Dis. (2019) 46(9):594–601. https://doi.org/10.1097/OLQ.0000000000001031.

SUPPLEMENTARY MATERIAL - FIGURES
